# Different bacterial gene expression patterns and attenuated host immune responses are associated with the evolution of low-level vancomycin resistance during persistent methicillin-resistant *Staphylococcus aureus *bacteraemia

**DOI:** 10.1186/1471-2180-8-39

**Published:** 2008-02-27

**Authors:** Benjamin P Howden, Danielle J Smith, Ashley Mansell, Paul DR Johnson, Peter B Ward, Timothy P Stinear, John K Davies

**Affiliations:** 1 Australian Bacterial Pathogenesis Program, Department of Microbiology, Monash University, Clayton, Victoria, Australia; 2 Infectious Diseases Department, Austin Health, Heidelberg, Victoria, Australia; 3 Australian Research Council Centre of Excellence in Structural and Functional Microbial Genomics, Monash University, Clayton, Victoria, Australia; 4 Centre for Functional Genomics and Human Disease, Monash Institute of Medical Research, Monash University, Clayton, Victoria, Australia; 5 Microbiology Department, Austin Health, Heidelberg, Victoria, Australia

## Abstract

**Background:**

Low-level vancomycin resistance in *Staphylococcus aureus *(vancomycin-intermediate *S. aureus *(VISA) and hetero-VISA [hVISA]) emerges during persistent infection and failed vancomycin therapy. Up-regulation of genes associated with the "cell wall stimulon" and mutations in the *vraSR *operon have both been implicated in the development of resistance, however the molecular mechanisms of resistance are not completely understood. To further elucidate the mechanisms leading to resistance transcriptome comparisons were performed using multiple clinical pairs of vancomycin-susceptible *S. aureus *(VSSA) and hVISA/VISA (n = 5), and three VSSA control pairs from hospitalized patients with persistent bacteraemia that did not develop hVISA/VISA. Based on the transcriptome results multiple genes were sequenced and innate immune system stimulation was assessed in the VSSA and hVISA/VISA pairs.

**Results:**

Here we show that up-regulation of *vraS *and the "cell wall stimulon" is not essential for acquisition of low-level vancomycin resistance and that different transcriptional responses occur, even between closely related hVISA/VISA strains. DNA sequencing of *vraSR*, *saeSR*, *mgrA*, *rot*, and *merR *regulatory genes and upstream regions did not reveal any differences between VSSA and hVISA/VISA despite transcriptional changes suggesting mutations in these loci may be linked to resistance in these strains. Enhanced capsule production and reduced protein A expression in hVISA/VISA were confirmed by independent bioassays and fully supported the transcriptome data. None of these changes were observed in the three control pairs that remained vancomycin-susceptible during persistent bacteremia. In a macrophage model of infection the changes in cell surface structures in hVISA/VISA strains were associated with significantly reduced NF-κB activation resulting in reduced TNF-α and IL-1β expression.

**Conclusion:**

We conclude that there are multiple pathways to low-level vancomycin resistance in *S. aureus*, even among closely related clinical strains, and these can result in an attenuated host immune response. The persistent infections associated with hVISA/VISA strains may be a consequence of changes in host pathogen interactions in addition to the reduced antibiotic susceptibility.

## Background

Low-level vancomycin resistance in *Staphylococcus aureus *(vancomycin-intermediate *S. aureus *[VISA], and heterogenous-VISA [hVISA]) is associated with vancomycin treatment failure and persistent infection [1-4]. The mechanisms underlying hVISA/VISA appear to involve step-wise processes under vancomycin selective pressure [5,6]. Global metabolic changes affecting the synthesis and structure of the bacterial cell wall are the major phenotypic features. These include cell wall thickening [7-9] associated with increased production of abnormal muropeptides [10], increased D-ala-D-ala residues and reduced peptidoglycan cross-linking [11], reduced growth rate, and reduced autolytic activity [8,11,12]. The thickened cell wall that results is thought to prevent diffusion of vancomycin to its active site in the cytoplasmic membrane [13].

The two-component regulatory system *vraSR*, was shown to be up-regulated in the Japanese strains Mu50 and Mu3 [14] and the VISA strain JH9 [15]. *vraSR *is a part of the "cell wall stimulon" that positively modulates cell wall biosynthesis, is induced by cell wall active agents [16,17], and has been suggested to be consistently up-regulated in VISA strains [15,18]. It is postulated that the cell wall active glycopeptides stimulate the over expression of the "cell wall stimulon" and promote resistance by increasing cell wall thickness. Recently, the complete genome sequencing of an isolate pair from a single patient demonstrated a small number of mutations associated with the VISA phenotype. Early in the evolution of resistance a mutation in the *vraSR *operon was detected [6]. Point mutations and inactivation of *agr*, an important quorum sensing global regulatory system in *S. aureus*, have been previously described in some VISA strains [19], and were associated with higher levels of resistance in strain JH9 [6], but are not a consistent finding. Recently, a number of additional genes were found to be over expressed in VISA strains and selective over-expression of these genes in the vancomycin susceptible strain N315 led to subtle increases in vancomycin resistance [20]. Most studies to date have been performed with *in vitro *or *in vivo *laboratory derived strains and the relevance of these findings to clinical infection in humans is unclear. A major limitation of previous studies assessing transcriptional changes in hVISA/VISA strains is that only single pairs of isolates have been used. This has made it difficult to draw conclusions about the relevance of the transcriptional differences in VISA strains as often hundreds of genes were differentially regulated [15,17,21].

We recently characterized five clinical pairs of VSSA and hVISA/VISA, isolated from patients with persistent MRSA bacteremia, and in whom vancomycin treatment failed [12]. These isolates demonstrated phenotypic changes commonly associated with VISA strains, including cell wall thickening and reduced autolytic activity. The level of resistance demonstrated in these strains is more frequently found clinically, as strains with a vancomycin MIC of ≥ 8 mg/L are still uncommon. In addition, we have obtained three pairs of clinical isolates from patients with persistent MRSA bacteremia and vancomycin treatment failure, in whom low-level vancomycin resistance did not develop. We used microarray analysis to investigate the transcriptional differences in the five VSSA and hVISA/VISA pairs and the three control pairs to determine if consistent transcriptional changes occur in clinical MRSA isolates associated with persistent bacteraemia and a common level of vancomycin resistance (hVISA/VISA with vancomycin MICs of 2–4 mg/L). Because many patients infected with hVISA/VISA strains have persistent infection, and because of the significant changes in surface structure of these hVISA/VISA strains, the impact of the VISA/VISA phenotype on host-pathogen interactions was also assessed.

Here we demonstrate that transcriptome patterns are divergent in different isolates pairs, and up-regulation of the "cell wall stimulon" and mutations in *vraSR *are not essential for the expression of vancomycin resistance in hVISA/VISA isolates. In addition we demonstrate for the first time that the hVISA/VISA phenotype is associated with significant changes in the expression of capsule and protein A on the cell surface, and hVISA/VISA strains produce less innate immune system activation.

## Results

### Isolate characteristics

Five VSSA and hVISA/VISA isolate pairs (pairs 1 to 5) were included in this study as well as 3 control pairs of strains (pairs 6 to 8). The reason for including the control pairs was to determine if transcriptional changes are found in MRSA isolates after persistent bacteraemia, but where hVISA/VISA does not develop. If similar transcriptional changes were found in the control strains as in the VSSA and hVISA/VISA pairs it would suggest these changes were independent of the resistance mechanism. In the three control pairs (pairs 6 to 8) pulsed field gel electrophoresis (PFGE) demonstrated identical banding patterns for isolate pairs, and identical *spa *types (JKD6084 and JKD6089, *spa *type 382; JKD6090 and JKD6094, *spa *type 382; JKD6095 and JKD6097, *spa *type 3). All 6 control isolates were fully vancomycin susceptible by population analysis profile testing, and had vancomycin MICs within the susceptible range (Table 1).

**Table 1:** Study isolates and susceptibility results

**Isolate**	**Phenotype**^a^	**MIC^b^(μg/ml)**		**Reference or Comment**
			
		**VCM**	**TEIC**	
**Pair 1**				
JKD 6000	VSSA	2.0	0.5	[12]
JKD 6001	VISA	4.0	8.0	[12]
**Pair 2**				
JKD 6009	VSSA	1.0	0.5	[12]
JKD 6008	VISA	4.0	2.0	[12]
**Pair 3**				
JKD 6021	VSSA	1.0	0.25	[12]
JKD 6023	VISA	4.0	8.0	[12]
**Pair 4**				
JKD 6052	VSSA	1.0	0.5	[12]
JKD 6051	hVISA	2.0	4.0	[12]
**Pair 5**				
JKD 6004	VSSA	1.0	0.5	[12]
JKD 6005	hVISA	2.0	4.0	[12]
**Pair 6 (control)**				
JKD 6084	VSSA	0.5	≤ 0.25	[this study]
JKD 6089	VSSA	1.0	≤ 0.25	[this study]
**Pair 7 (control)**				
JKD 6090	VSSA	0.5	≤ 0.25	[this study]
JKD 6094	VSSA	0.5	≤ 0.25	[this study]
**Pair 8 (control)**				
JKD 6095	VSSA	1.0	0.5	[this study]
JKD 6097	VSSA	1.0	0.5	[this study]
Other Isolates				
P1				Cap8 positive [57]
Newman				Cap5 positive [57]

^a^VSSA, vancomycin-susceptible *S. aureus*; VISA, vancomycin-intermediate *S. aureus*; hVISA, hetero-VISA.

^b^Minimum inhibitory concentration. VCM, vancomycin; TEIC, teicoplanin.

### Microarray analysis of control pairs

No genes were differentially expressed in pairs 7 and 8 when the later clinical isolate (JKD6094 and JKD6097) were compared to the earlier isolate (JKD6090 and JKD6095). For isolate pair 6 only two genes were differentially expressed; SACOL0935 (D-alanine-activating enzyme/D-alanine-D-alanyl carrier protein ligase), fold ratio 2.32; and SACOL0936 (DltB, a putative activated D-alanine membrane transport protein), fold ratio 2.27.

### Microarray analysis of clinical hVISA/VISA strains

**Figure 1:**
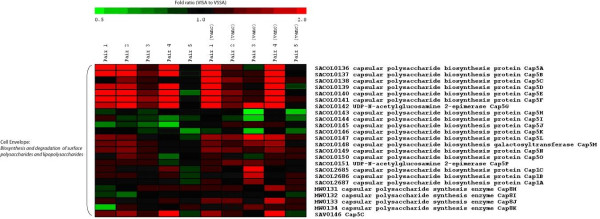
**Heat map analysis of selected genes**. Heat map analysis of selected genes which were differentially expressed in at least 2 isolate pairs. The results are expressed as fold ratio of gene expression for hVISA/VISA compared to VSSA. Results are presented for all 5 isolate pairs without antibiotic exposure and all 5 isolate pairs after exposure to vancomycin. This figure shows the upper quartile of these selected genes, for the full image please see additional file 1.

For the VSSA and hVISA/VISA pairs a list of genes of interest was generated as described in the methods section. These results are presented as the fold ratio of hVISA/VISA compared to the related VSSA isolate. There were 54 genes that were down-regulated, and 89 genes that were up-regulated in at least two isolate pairs. Selected genes from this list which were of particular interest are highlighted in figure 1 and additional file 1. The complete gene list is also provided in additional file 2. Down-regulation of the quorum sensing global regulator *agr *(*agrA*, *agrB*) was prominent across three pairs. This would be expected to have secondary effects on exotoxin production and expression of cell adhesion molecules, and a number of genes involved in pathogenesis and toxin production, and cell surface adhesion molecules were indeed significantly down-regulated (*spa*, *fnbA*, *fnbB*, *efb*, putative exotoxin SACOL0478, fibrinogen-binding protein related proteins and precursors SACOL1164 and SACOL1169). Of particular note, marked down-regulation of the *spa *gene SACOL0095 was demonstrated in four of the five hVISA/VISA isolates, and another IgG-binding protein, SACOL2418 was down-regulated in all five isolates.

A number of contiguous genes were up-regulated. This included genes involved in urease production and nitrogen metabolism (*ureA, ureB, ureC, ureD, ureE, ureF, ureG, narG, narH, nirD*) and amino acid biosynthesis for aspartate (*hom*, *lysC, asd, dapA, dapB, dapD*). In addition genes encoding pyruvate biosynthesis were also up-regulated (*ilvB, ilvC, ilvE, leuA*). Of particular interest was the up-regulation of capsular polysaccharide biosynthesis genes (*cap5A*, *cap5B*, *cap5D*, *cap5E*, *cap5F*) in a number of hVISA/VISA isolates. The gene encoding the SceD protein (SACOL2088, a putative transglycosylase) was frequently up-regulated. Regulators other than *agr*, which had differential expression, included the *merR *family regulators SACOL2193 and SACOL2517 in 4 and 3 pairs respectively, the GntR family regulator (SACOL1997) in 2 pairs, and up-regulation of a putative regulatory gene (SACOL2585) in 2 pairs, but down-regulation in another pair.

Previous studies have demonstrated significant transcriptional changes in *S. aureus *isolates when compared to non-exposed isolates, after exposure to vancomycin. We therefore exposed both the susceptible and hVISA/VISA isolate in pairs 1 to 5 to vancomycin for 30 min prior to RNA extraction and microarray analysis, in an effort to detect differences in responses to vancomycin exposure in hVISA/VISA and VSSA isolates from the same pair. After exposure of the VSSA and hVISA/VISA in each pair to vancomycin there was a more pronounced gene down-regulation compared to the initial analysis (Figure 1, and additional file 2). This included significant down-regulation across 3 of the 5 pairs in the 2-component system *saeSR*, and marked down-regulation of MW2407 encoding a hypothetical protein in multiple pairs, suggesting that vancomycin led to differential effects in gene regulation between the hVISA/VISA isolate and VSSA in each pair. There were however, no consistent effects of the vancomycin exposure across all isolate pairs.

A heat map analysis was also performed to assess global changes in all VSSA and hVISA/VISA isolate pairs, using the complete microarray data sets. The heat map of all microarray data (additional file 3) demonstrates that although there are some consistent changes across a few of the isolate pairs (in a small subset of genes, such as genes encoding capsule production, protein A, and urease production), there are significant differences in the global transcriptional patterns in different hVISA/VISA isolates, such that the global transcriptional patterns were divergent between different isolate pairs.

### Genes associated with the "cell wall stress stimulon" are not consistently up-regulated in hVISA/VISA

**Figure 2:**
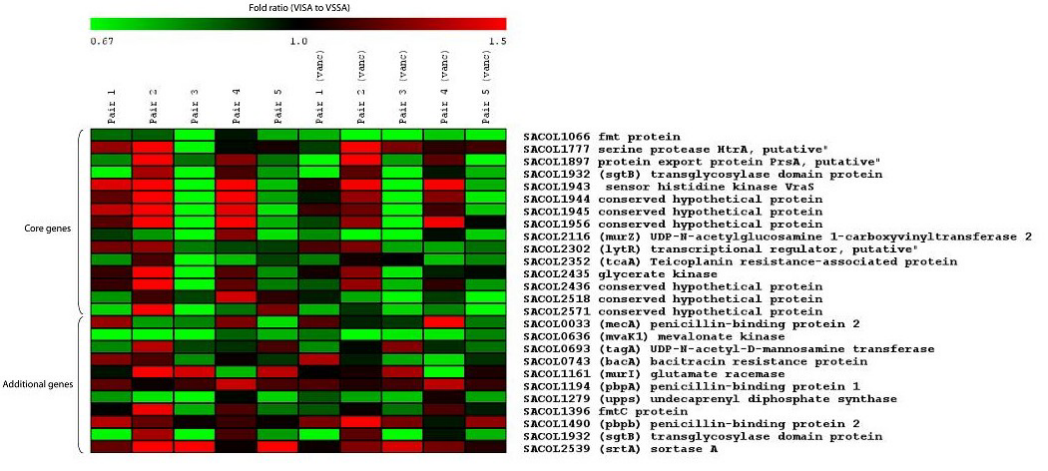
**Heat map analysis of cell wall genes**. Heat map analysis of "core cell wall genes" and additional cell wall genes which have been commonly shown to be important in cell wall biosynthesis [56]. The results are expressed as fold ratio of gene expression for hVISA/VISA compared to VSSA. Results are presented for all 5 isolate pairs without antibiotic exposure and all 5 isolate pairs after exposure to vancomycin.

Because of the recent interest in the importance of up-regulation of the "cell wall stimulon" in hVISA/VISA, linked with cell wall thickening and resistance, we specifically analyzed the transcriptional changes in these genes. Two isolates pairs demonstrated up-regulation of *vraS *and associated genes in the initial microarray analysis, but of particular interest was the lack of any significant change in two pairs (pair 1 and 5), and the down regulation in the another pair (pair 3) (Figure 2). After vancomycin exposure a number of these cell wall genes were also down regulated in pair 5 (SACOL1066, SACOL1943, SACOL1944, SACOL2116, SACOL2571).

### Protein expression and capsule production correlate with array results

**Figure 3:**
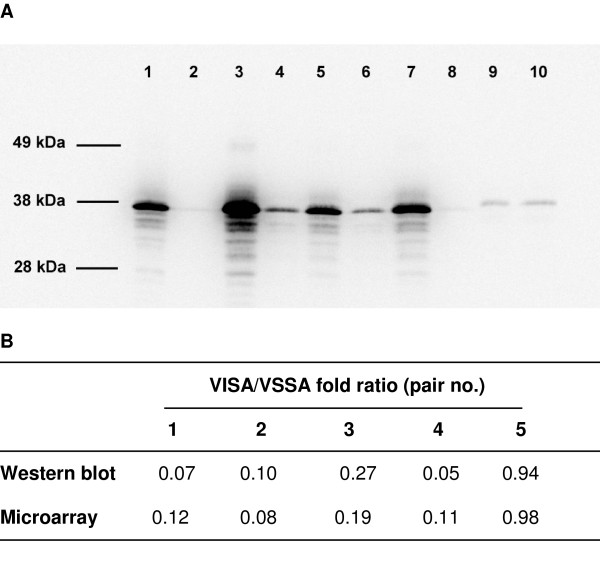
**Western blot analysis of protein A production**. Analysis of protein A production by Western Blot in hVISA/VISA and VSSA pairs. A. Pair 1 (lane 1, JKD 6000; lane 2, JKD 6001), pair 2 (lane 3, JKD 6009; lane 4, JKD 6008), pair 3 (lane 5, JKD 6021; lane 6, JKD 6023), pair 4 (lane 7, JKD 6052, lane 8, JKD 6051), pair 5 (lane 9, JKD 6004; lane 10, JKD 6005). B. Fold ratios for protein A production and protein A (*spa*) gene expression for 5 hVISA/VISA and VSSA pairs.

**Figure 4:**
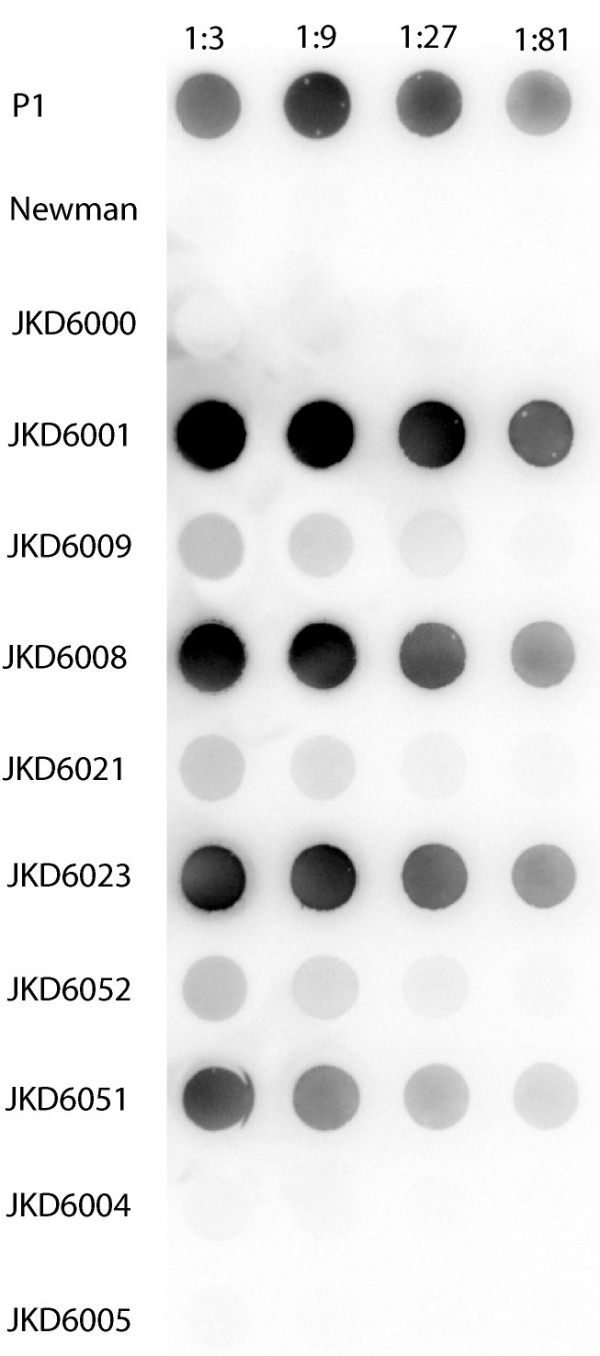
**Capsule immunoblot comparison of VSSA to hVISA/VISA**. Capsule immuoblot using anti-capsule type 8 antibody. Serial 3-fold dilutions of crude capsule extracts were loaded onto nitrocellulose membrane. Positive control, capsule type 8 positive strain P1; negative control, capsule type 5 positive strain Newman.

To determine if the transcriptional changes that were detected in hVISA/VISA isolates resulted in changes in protein A and capsule production, as well as urease activity, expression was determined for all isolates. A significant decrease in protein A production was demonstrated for 4 of the 5 pairs (Figure 3). For the 5^th ^pair, protein A production was low in both isolates. Urease activity was higher for the hVISA/VISA isolates compared to the VSSA isolates for 4 of the 5 pairs (Table 2). These results correlated well with *spa *and *ureA *gene expression (Table 2, Figure 3, additional file 2). A PCR was designed to determine the capsule genotype of isolates (capsule type 5 or capsule type 8). All isolates from pairs 1 to 5 were capsule type 8 strains by PCR. Capsule immunoblot confirmed the PCR capsule typing results, and demonstrated a significant increase in capsule expression in 4 of the 5 pairs (Figure 4). The last pair (no 5) had minimal capsule expression in the VSSA or hVISA strain, and these results also correlated well with microarray data.

**Table 2:** Urease assay results and comparison to microarray results

		**VISA/VSSA fold ratio**	
		
**Strain**	**Urease activity (μmol urea min^-1 ^mg protein^-1^)**	**urease assay**	**microarray (*ureA*)**
**Pair 1**			
JKD 6000	0.0		
JKD 6001	8.2	>8.2	3.75*
**Pair 2**			
JKD 6009	3.9		
JKD 6008	6.8	1.7	1.40
**Pair 3**			
JKD 6021	3.7		
JKD 6023	17.1	4.6	1.82*
**Pair 4**			
JKD 6052	3.8		
JKD 6051	5.17	1.5	2.69*
**Pair 5**			
JKD 6004	2.3		
JKD 6005	1.5	0.65	0.73

**Abbreviations**: VSSA, vancomycin-susceptible *S. aureus*; VISA, vancomycin intermediate *S. aureus*.

*Microarray fold ratios were statistically significant.

### Sequencing of regulatory genes does not demonstrate mutations leading to altered regulatory gene expression

In an effort to identify any genetic mutations leading to the resistant phenotype in the 5 hVISA/VISA strains, regulatory loci which were differentially expressed in the microarray experiments or which were predicted to be linked to the microarray results were sequenced in all 5 VSSA and hVISA/VISA pairs. We found no sequence changes between the VSSA and hVISA/VISA in each pair in the two-component regulators *saeSR *and *vraSR*, nor in the genes encoding the *merR*-like regulators SACOL2193 and SACOL2517. The regulatory genes *mgrA *(SA0641) and *rot *(repressor of toxins) were also sequenced. Although *mgrA *was not differentially expressed on the microarray results, it has recently been shown to be an important global regulator in *S. aureus*, with effects on autolytic activity, protein A expression and capsule production [22]. r*ot *is a *sarA *homologue which is blocked by RNAIII [23,24]. It has effects on expression of multiple genes, including those involved in synthesis of urease, protein A, and ABC transporters [25]. Again, no mutations were found in either gene. Finally, because of the dramatic decrease in MW2407 expression in the vancomycin exposure experiments, the region MW2406 to MW2408 was sequenced (this gene on the TIGR version *S. aureus *array is unique to MW2). The expected product size based on the MW2 genome was 2898 bp, but the amplified fragment from our strains was ~1900 bp. There were no differences in the sequences between the VSSA and hVISA/VISA strains. The region amplified in our strains matched closely to 2 regions in MRSACOL encoding tandem lipoproteins and a hypothetical protein (SACOL2496, SACOL2498 and SACOL2492, SACOL2493).

### Increasing vancomycin resistance is associated with reduced NF-κB activation and reduced pro-inflammatory cytokine release

**Figure 5:**
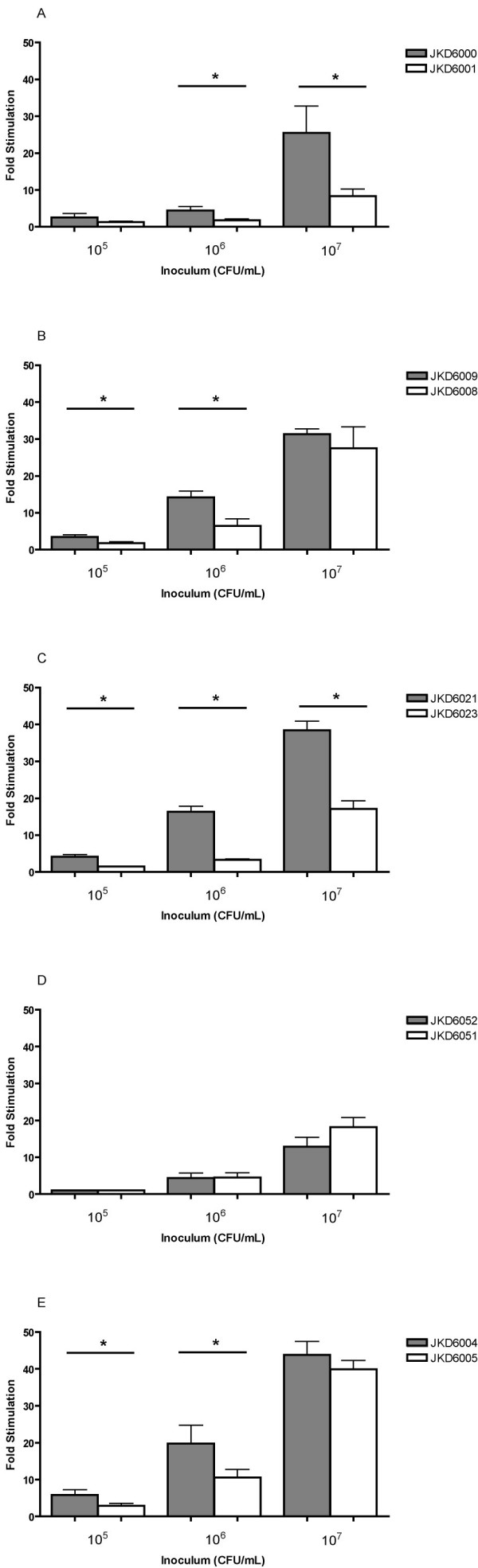
**NF-κB activation results for VSSA and hVISA/VISA isolate pairs**. Stably transfected RAW cells with an ELAM-NF-kB reporter construct were exposed to formaldehyde killed VSSA and hVISA/VISA pairs for 6 hours. Results are presented as fold NF-κB activation for hVISA/VISA compared to VSSA and are the result of multiple replicates. (* *p *< 0.05)

**Figure 6:**
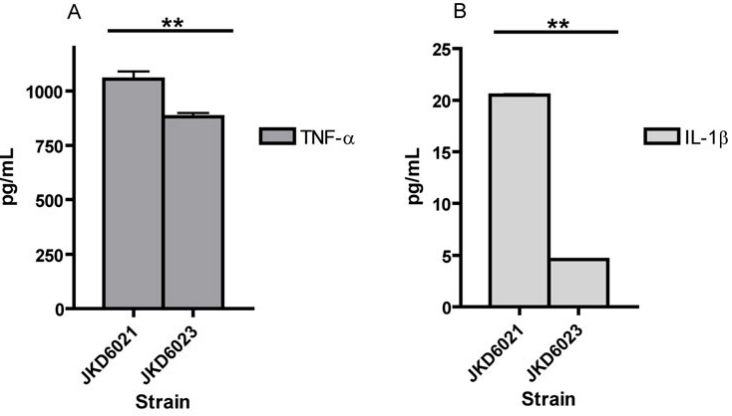
**TNF-α and IL-1β expression**. TNF-α and IL-1β expression from RAW264.7 cells after 6 hours exposure to formaldehyde killed JKD6021 (VSSA) and JKD6023 (VISA). (** *p *< 0.001).

Because we detected a number of significant changes in staphylococcal surface structures that interact with the innate immune system during the evolution of hVISA/VISA, we next wished to establish if hVISA/VISA displayed a decreased activation of the prototypic inflammatory transcription factor NF-κB. As shown in Figure 5, there was a statistically significant decrease in NF-κB activation demonstrated with 4 of the 5 hVISA/VISA isolates compared to their parent VSSA strain, particularly at lower concentrations. This appeared to correlate with the vancomycin MIC result, with reduced NF-κB activation detected with all the strains with a vancomycin MIC of 4 mg/L, but only with one of those with an MIC of 2 mg/L. To determine the biological impact of this effect, a single pair (JKD6021 and JKD6023) were selected to determine if the reduced NF-κB activation resulted in a diminished expression of the NF-κB-dependent inflammatory cytokines TNF-α and IL-1β. As demonstrated in Figure 6, after 6 hours exposure to both strains, there was a significant decrease in TNF-α expression when comparing JKD6021 to JKD6023 (p < 0.001), while IL-1β expression was substantially decreased.

## Discussion

Methicillin-resistant *Staphylococcus aureus *(MRSA) is on the rise globally [26,27], with recent failures of vancomycin therapy for MRSA infections associated with low-level resistance to glycopeptides [3,28]. In addition, persistent MRSA bacteremia despite vancomycin therapy has also been recognized as an important clinical syndrome which is incompletely understood [29]. We have investigated transcriptional changes in five clinical pairs of VSSA and hVISA/VISA isolated from patients with persistent MRSA bacteremia on vancomycin therapy. Multiple gene expression changes were found in the hVISA/VISA strains compared to the parent VSSA, but essentially no changes were found in three control pairs of strains, suggesting the transcriptional changes in the hVISA/VISA strains are linked to resistance. Despite the different PFGE patterns [12], the five isolate pairs are closely related as determined by sequence based typing (by multi-locus sequence typing all are ST239-MRSA) [data not shown] and 4 of the 5 isolate pairs have identical *spa *types [12]). Although there were some consistent transcriptional changes found, there were very few consistent changes across all pairs, suggesting that multiple transcriptional pathways lead to low-level vancomycin resistance in *S. aureus*, and this is further supported by the detailed analysis of "cell wall stimulon" activation in our strains. These results are most clearly demonstrated by the global heat map analysis (additional file 3) that displays the major differences in the transcriptional patterns among the isolate pairs. In addition, we have demonstrated for the first time that the development of low-level vancomycin resistance in patients with persistent bacteraemia is associated with changes in the cell surface and alterations in the host immune response. These are important findings demonstrating that *S. aureus *strains evolve to alter immune system activation, in association with changes leading to reduced response to vancomycin therapy.

A consistent theme from other studies of VISA strains has been the up-regulation of cell wall biosynthetic genes [14,15,18,21], including the *vraSR *two-component regulatory system and genes from this regulon. It is hypothesized that vancomycin activates the "cell wall stress stimulon" which then remains activated in VISA strains even after the vancomycin is removed. However, by examining multiple clinical isolates, we have established that low-level vancomycin resistance can be acquired in *S. aureus *without up-regulation of *vraSR *and other genes of the "cell wall stimulon". In fact, these genes were down-regulated in one VISA strain (JKD6023) in the initial analysis and in another strain (JKD6005) after vancomycin exposure. This is an important finding that clearly demonstrates that the hVISA/VISA phenotype can develop in clinical *S. aureus *isolates (associated with cell wall thickening and reduced autolytic activity [12]) without a sustained induction of the "cell wall stimulon". In addition, despite recent data suggesting that mutations in the *vraSR *operon are common in VISA strains [6], we found no mutations in this region in our strains. It is important to note that our hVISA/VISA isolates are all the same MLST type, and therefore are not from significantly different genetic backgrounds. This suggests that different pathways to resistance exist, even in genetically closely related strains, but that our results may not however apply to strains of other genetic backgrounds, such as Mu50 and Mu3.

Recent studies have highlighted some of the difficulties in interpreting microarray data without analysis of the effects the transcriptional changes on the expression of gene products [30]. We addressed this issue by quantitating a number of the gene products which were differentially expressed in the hVISA/VISA isolates. The microarray data was supported by protein A production, capsule expression and urease activity analysis. In addition, our previous real-time PCR analysis of RNAIII expression correlates well with the decreased *agr *expression found in the microarray analysis [12]. The increased capsule expression and reduced protein A production demonstrated in 4 of the 5 hVISA/VISA strains is of interest. These changes are likely to have significant implications for host pathogen interactions, and in fact, could favor persistent infection. For this reason, we performed experiments to determine the impact of the hVISA/VISA phenotype on immune system activation, as measured by NF-κB activation.

We have clearly demonstrated that the development of VISA is associated with changes in NF-κB activation, which will affect the host pro-inflammatory response to infection. This was clearly demonstrated with the 3 VISA strains that had a vancomycin MIC of 4 mg/L, and one of the strains with a vancomycin MIC of 2 mg/L (Figure 5). Although hVISA/VISA strains may have slower growth rates compared to VSSA, we controlled for this in our NF-κB activation assays by growing cells to stationary phase and performing colony counts so that equivalent inoculums of killed cells were used in the assays. Many different staphylococcal components activate the innate immune system and induce NF-κB activation; including protein A, teichoic acids and peptidoglycan [31]. The activation of NF-κB in turn up-regulates the expression of pro-inflammatory cytokines including TNF-α and IL-1β. The biological impact of this altered immune response was further highlighted by using the strain pair JKD6021 and JKD6023 which confirmed that RAW cells exposed to VISA strains compared to VSSA isolates display a reduced TNF-α and IL-1β expression. Secreted exotoxins also activate innate immune responses, and although we did not measure these directly, the down-regulation of *agr *in hVISA/VISA strains, including our strains, would also limit immune activation by reducing exotoxin production. The cell surface changes we demonstrated in our hVISA/VISA isolates more closely associate to the altered host responses demonstrated here.

Our results confirm the potential importance of changes in the *agr *two-component regulatory system in hVISA/VISA strains [32,33]. Recently, reduced *agr *function has been demonstrated in isolates from patients with persistent MRSA bacteraemia where vancomycin resistance was not demonstrated [29,34]. Although we have demonstrated reduced *agr *expression in a number of our strains previously by real time PCR [12] and now by microarray analysis, we did not find any changes in *agr *expression in isolates from patients with persistent bacteremia without hVISA/VISA. This suggests that there is a link between reduced *agr *expression and low-level vancomycin resistance, but that reduced *agr *expression is not always associated with prolonged MRSA bacteraemia and vancomycin therapy as previously suggested [29,34].

Although up-regulation of capsule genes has been demonstrated in other VISA strains [15,20] enhanced capsule production has never previously been confirmed. Loss of capsule typability has been reported in VISA revertant strains obtained by serial passage [35], and electron microscopy of VISA strain JH9 demonstrates what is probably excess capsule [11], however the potential importance of capsule expression in the hVISA/VISA phenotype has not been considered. Over-expression of staphylococcal capsular polysaccharide type 8 has been shown to protect against *in vitro *opsonophagocytic killing by human neutrophils and lead to more persistent blood stream and organ infection in mice [36]. Recently, up-regulation of capsule gene expression has been documented as part of the global transcriptional response that contributes to innate immune system evasion [37]. It is conceivable that the changes in capsule expression are an adaptation to allow the bacteria to persist in the blood stream and evade immune system killing. It has also been shown that *S. aureus *with increased capsule are less adherent to epithelial cell monolayers, and to damaged heart valves [38], and recently is has been determined that increased capsule expression blocks clumping factor A-mediated binding of staphylococci to fibrinogen and platelets [39]. Therefore the increased capsule changes we observed may play a role in the reduced biofilm formation that we have previously demonstrated in these same strains [12], possibly by masking important adhesins involved in the early stages of biofilm formation. It is also worth considering if the increased capsule production could directly contribute to the low-level vancomycin resistance in some hVISA/VISA strains, or interfere with the *agr *quorum sensing system, and explain the quorum sensing changes observed in these hVISA/VISA strains (reduced *agr *expression), especially as mutations in the *agr *operon were not found in the strains [12]. More work is needed to determine if increased capsule expression is common in many hVISA/VISA strains, and to understand the impact of capsule production on the expression of low-level vancomycin resistance in *S. aureus*, and on the *agr *system.

Down-regulation of *spa *is one of the most consistent transcriptional changes found in hVISA/VISA strains [14,15,17,33,40]. We have shown by western blot that reduced *spa *expression leads to almost complete absence of protein A expression in hVISA/VISA strains. In addition, consistent down-regulation of the gene encoding another IgG-binding protein (Sbi) was also found. These changes are likely to have significant biological implications, and could partly explain the changes in NF-κB activation. Protein A is a major surface protein of *S. aureus*. It binds to the Fc region of IgG, and the Fab portion of Ig belonging to the VH3+ gene family [41] where it may act as a B cell superantigen, but also binds to several other proteins including gC1qR which is expressed on activated platelets [41]. Protein A triggers platelet aggregation [42], and along with other staphylococcal surface proteins (such as fibronectin binding proteins [FnBPs]) has been shown to activate platelets [43,44]. Upon activation platelets release platelet microbiocidal proteins (PMPs) [45]. The interaction between *S. aureus *and platelets, and in particular the role of PMPs in protection against intravascular infections, and the link between hVISA/VISA and reduced susceptibility to PMP has been well described [29,32]. The decreased protein A and FnBPs expression seen in our hVISA/VISA strains would be expected to lead to less platelet activation. Recently, protein A has also been shown to activate a respiratory epithelial inflammatory response by binding to TNR-α receptor 1 [46]. Staphylococcal protein Sbi is an IgG- and β_2 _glycoprotein I-binding protein that has been found to be up-regulated after exposure to human serum [47]. Decreases in *spa *expression were not seen in the 3 control pairs of strains. Nonetheless, along with increased capsule expression the *spa *changes have the potential to alter immune system response to infection and promote persistent infection.

Increased urease activity has not been reported in hVISA/VISA strains. Urease is involved in the urea cycle and amino acid metabolism as well as purine metabolism [48]. The reasons for the increased production of urease in our hVISA/VISA strains is not clear, but may be linked to increased amino acid biosynthesis, with increased expression of genes involved in aspartate biosynthesis in particular found in the microarray analysis. Recently, up-regulation of urease production has been demonstrated in *S. aureus *biofilms, and it was hypothesized that increased ammonium and/or ammonia is required to counteract the acid environment in the biofilm. It is not clear how this may be linked to the hVISA/VISA phenotype, except that increased energy requirements and increased metabolism in the hVISA/VISA cell may require increased urease activity.

Sequencing of a number of regulatory genes that were differentially expressed in the microarray analysis (*vraSR*, *saeSR*, SACOL2193, SACOL2517) or were potentially involved in the changes we found (*mrgA *and *rot*) revealed no nucleotide changes. However, any mutations associated with resistance in these strains are clearly leading to global transcriptional changes in hVISA/VISA isolates. The recent work by Mwangi et al [6] demonstrated that a small number of mutations could lead to an increase in vancomycin MIC up to 4 mg/L in the strain they studied. In our previous study of these VSSA and hVISA/VISA clinical isolates we demonstrated by PFGE a difference in banding patterns for the pairs JKD6000/JKD6001 and JKD6021/JKD6023 [12]. A genomic microarray comparison using the whole genome of MRSACOL and selected unique genes from *S. aureus *strains Mu50, MW2 and N315 (TIGR version 2 array) failed to reveal any genetic changes in these pairs [12]. Because the aims of this study were to determine transcriptional changes using the TIGR ver2 arrays, we have not been able to provide any further explanation for the difference in PFGE banding patterns. It is possible that loss of phage DNA could explain the changes, and is unlikely to contribute to the resistance phenotype, but confirmation would require a whole genome comparison.

## Conclusion

The emergence of hVISA/VISA from vancomycin-susceptible *S. aureus *can occur by different transcriptional pathways, even in closely related strains, and it appears that up-regulation of the "cell wall stimulon" and mutations in *vraSR *are not essential for the generation of hVISA/VISA. Changes in capsule production and protein A expression frequently accompany the altered antibiotic susceptibility in hVISA/VISA strains, and reduced innate immune activation may be another factor promoting persistence of infection. Further work is required to fully understand the impact of changes in immune recognition of hVISA/VISA strains. Identifying the mutation or mutations leading to resistance in our strains will require a global comparative genomics approach.

## Methods

### Bacterial strains, antibiotic susceptibility and molecular typing

*Staphylococcus aureus *strains are listed in table 1. All strains were clinical isolates from patients with persistent methicillin-resistant *S. aureus *bacteraemia (blood culture positive after > 7 days of vancomycin therapy) [12]. Isolate pairs 1 to 5 have been previously well characterized and represent VSSA and hVISA/VISA pairs [12]. Isolate pairs 6 to 8 are blood culture isolates (patients with bacteremia for 8–22 days) where hVISA/VISA was not detected in the later clinical isolate. All isolates were grown in brain heart infusion broth (BHIB) (Oxoid). For the control pairs vancomycin population analysis profile (PAP), vancomycin and teicoplanin broth minimum inhibitory concentrations (MIC), PFGE and *spa *typing were performed as previously described [12].

### Preparation of total RNA

After overnight growth in BHIB a 1 in 100 dilution was made in 50 ml of BHIB and incubated at 37°C with shaking. Exponential-phase culture (20 ml) was added to 10 ml of an RNA stabilization reagent (RNA *later*, Qiagen) and allowed to stand for 10 min after mixing. Total RNA was prepared using the RNeasy Midi Kit (Qiagen) [12]. For vancomycin experiments, vancomycin (Sigma) was added to cultures in early exponential growth (final concentration 0.5 × MIC) for 30 min before RNA extraction.

### Preparation of Cy3- and Cy5-dUTP labeled cDNA probe

Transcription profiling to compare the later clinical isolate (VISA/hVISA for pairs 1 to 5, VSSA for pairs 6 to 8) to the related initial clinical isolate (VSSA) was performed for each pair without antibiotics, and repeated after exposure of both the VSSA and hVISA/VISA isolates to vancomycin for pairs 1 to 5. cDNA was synthesized using SuperScript™ II RNase H-Reverse Transcriptase (Invitrogen) incorporating aminoallyl-dUTP at 42°C with the following reaction mixture (RNA 5 μg, random hexamers 6 μg, Superscript II RT 400 U, 0.5 mM dATP, dCTP, dGTP, 0.2 mM dTTP, 0.3 mM aa-dUTP [Amersham Biosciences]; total volume 30.7 μl). The RNA was hydrolyzed by addition of 10 μl 0.5 M EDTA and 10 μl 1 M NaOH with incubation at 65°C for 15 min, followed by addition of 25 μl 1 M Tris (pH 7.0) to neutralize the pH. The cDNA was collected using a QIAquick column (Qiagen) and dried to completion in a vacuum concentrator. The aminoallyl-labeled cDNA was resuspended in 4.5 μl 0.1 M sodium bicarbonate buffer (pH 9.3) and incubated at room temperature for 2 hours after addition of Cy3 or Cy5 (Amersham Biosciences) resuspended in 4.5 μl DMSO (Sigma). The probe was purified using a microcon-30 column. The quantity of cDNA and incorporation ratio of Cy dye was measured by spectrophotometry (NanoDrop ND-1000, NanoDrop Technologies, DE, USA) to confirm acceptable results and then the two differentially labeled probes were mixed and dried to completion in a vacuum concentrator.

### Array hybridization and analysis

Array hybridization, washing and scanning using version 2 *Staphylococcus aureus *microarray slides (The Institute for Genomic Research, MD, USA) was performed as described previously [12]. Briefly, 30 μl of hybridization mixture containing Cy3- and Cy5-dUTP labeled probe, 15 μl formamide (Sigma), 5 × SSC, 0.1% SDS, and 22.5 μg herring sperm DNA (Promega) was denatured at 95°C then applied to the microarray slide and incubated overnight at 42°C. The microarray was washed sequentially in 2 × SSC, 0.1% SDS; 0.1 × SSC, 0.1% SDS; and 0.1 × SSC and then dried. The microarrays were scanned with the GMS418 Array scanner (Genetic MicroSystems) to measure the fluorescence of the Cy3 and Cy5 labeled cDNA hybridized to the microarray. The images were combined and quantification of fluorescent and background intensity determined using ImaGene™ ver 5.1 (Biodiscovery).

RNA extraction and hybridizations were performed in triplicate, and the dye swapped with each replicate. Using BASE [49] the data from the three biological replicates were combined and analyzed using Bioconductor and in particular Limma [50]. After global lowess normalization the log-ratios for each array were scaled so that the median absolute deviation was the same between arrays. For each gene ID a fold ratio of later clinical isolate (hVISA/VISA for pairs 1 to 5) compared to the earlier clinical isolate (VSSA) was calculated. Using a moderated t-test P-values were calculated and adjusted for multiple testing using false-discovery-rate (FDR). A ≥ 1.5-fold change and P value < 0.05 was considered significant. For the hVISA/VISA comparison any genes which were consistently up or down regulated across at least two clinical pairs was included in the set of "genes of interest". Heat maps of the normalized microarray results were generated using MeV [51].

### Protein Extraction

Cell wall associated proteins for protein A quantification were extracted as previously described [52]. Isolates were grown in BHIB in an identical manner as described for RNA extractions. Thirty ml of exponential stage culture was placed in a falcon tube and centrifuged at 5000 × g for 10 min. After removal of the supernatant 600 μl of extraction buffer (30% raffinose in 0.05 M Tris [pH 7.5] with 0.145 M NaCl) containing lysostaphin (Sigma) (250 ug), 10 μg of DNase (Sigma), 1 mg ml^-1 ^iodoacetamide (Sigma), and 1 mM ml^-1 ^phenylmethylsulfonyl fluoride (Sigma) was added. The mixture was incubated for 1 h at 37°C with rotation and then centrifuged at 8000 × g for 10 min, and the supernatant was centrifuged at 8000 × g for a further 10 min. Cellular protein for assessment of urease activity was extracted from eight ml of culture. The culture was pelleted, washed in PBS at 4°C, and resuspended in 1 ml PBS (4°C). The cells were disrupted in a FastPrep™ FP120 (Bio101 Savant, CA, USA) (setting 6 for 45 sec × 2) and the tube was centrifuged at 12,000 × g for 30 min at 4°C. The supernatant was transferred to a new tube. Protein concentrations were measured using the BCA protein assay kit (Pierce, IL, USA) and stored in aliquots at -80°C.

### SDS-PAGE and western blot analysis for protein A expression

Fifteen μg of cell wall associated protein from each isolate was separated in a 12% (wt vol^-1^) SDS-PAGE gel. The protein samples were transferred from the SDS-PAGE gel to cellulose nitrate transfer membrane (Schleicher & Schuell). A mouse derived monoclonal anti-protein A antibody (2000 × dilution) (Sigma), and secondary sheep anti-mouse IgG peroxidase conjugate (2000 × dilution) (Silenus) were used. The blots were developed using the Western Lightening chemiluminescence kit (Perkin-Elmer) and the image acquired and analyzed using the LAS-3000 Luminescent Image Analysis System (Fujifilm, Tokyo, Japan) and Multi Gauge version 2.2 software (Fujifilm, Tokyo, Japan).

### Urease Assays

Urease activity was measured using a coupled enzyme assay [53]. The 1.5 ml reaction mixture contained 31 mM Tris-HCl (pH 8.0), 10 mM urea (Amresco, OH, USA), 810 μM 2-oxoglutarate (Sigma), 240 μM NADH and 1.34 mg glutamate dehydrogenase (Sigma). Reactions were performed at room temperature and NADH degradation was measured by absorbance at 340 nM using a 552 UV-VIS spectrophotometer (Perkin-Elmer). The urease activity of each isolate was expressed as μmol of urea hydrolyzed min^-1 ^mg of protein^-1^.

### Capsule typing and Quantification

A multiplex PCR for capsule genotyping (CP5 and CP8) was designed using sequences available in NCBI (accession numbers for cap5, U811973; cap8, U73374). The forward primer was in Cap5/8G and the reverse primers in the variable regions, Cap5H and Cap8H (Table 3). The CP8 positive strain P1 and the CP5 positive strain Newman were used as controls. For capsule quantification a capsule immunoblot was performed [36]. Crude capsule extracts were prepared after growth in BHIB for 18 h at 37°C, using a previously described method [54]. After adjusting the OD600 to 0.5, 10 ml of culture was pelleted, resuspended in 500 μl PBS, then treated with the following enzymes consecutively at 37°C; lysostaphin (sigma) 200 μg ml^-1 ^for 15 min; DNase I (sigma) 300 U ml^-1 ^for 15 min; and proteinase K (sigma) 100 μg ml^-1 ^for 1 h, and then inactivated at 75°C for 10 min. Serial dilutions were loaded onto nitrocellulose membrane using a dot-blot apparatus. After blocking with 5% skim milk the membrane was incubated with CP8-specific rabbit antiserum kindly provided by Jean Lee, and then sheep anti-rabbit IgG peroxidase conjugate (Chemicon, Australia). The blots were developed as described above.

**Table 3:** Primers used in study

*mgrA *F	TTG AAG CAC ATG CAG AAA CA
*mgrA *R	TCG CAA CAA ACA CAA CCA TT
	
mw2406 to 2408 F	CCC AAT CTC CTT GAG CTA CAT T
mw2406 to 2408 R	TTA GTT GGT TAG GCC AAT AAA AA
	
*saeSR *F	GGC GGC ATA CAG TTA ATT TCA
*saeSR *R	CAC TCA TTG TTA AAA CAG ATT TCA CTT
	
*vraSR *F	TTG TCG GTG CTG AAA TCA AT
*vraSR *R	GTT GCG ACG GAT GAG GTT AT
	
*rot *F	TGT AGA ATT GTTGCA ATT TAA TGG T
*rot *R	TGC CAA CAA CAA AAA GAG GTT
	
SACOL2193 L	CCT TGA CCT ACT TCA GTT TCA TTT
SACOL2193 R	ATA AGA AGT ATT CAA ACG AAG ATG ACA
	
SACOL2517 L	GCG CTT TCT TTA CGA GCA CT
SACOL2517 R	TGA CGT CGG GTC ATC AAC TA
	
cap5/8G L	TTT TGA AGT TCC CTG GTG TCC
cap8H R	TAG CGC CAA GAA TCG CTA TCC
cap5H R	ACC AAC AAC CTC ATA TGC TCC

### DNA techniques

Genomic DNA was extracted using the GenElute Bacterial Genomic DNA Kit (Sigma). PCR was carried out using *Taq *DNA polymerase (Roche Molecular Biochemicals). DNA sequencing was performed using the BigDye Terminator version 3.1 cycle sequencing kits (Applied Biosystems) and the reaction mixtures were analyzed with the 3730 DNA Analyser (Applied Biosystems). Primers for amplifying and sequencing *vraSR, saeSR*, *rot*, SACOL2193 and SACOL2517, MW2406 to MW2407, and *mgrA *(SA0641) were designed using the MRSA COL, MW2 and N315 genome sequences (Table 3).

### NF-κB assay

*Staphylococcus aureus *cells were prepared from stationary phase cultures by washing in cold PBS and resuspending in 5% formaldehyde for 1 h, followed by additional washes in PBS. Colony counts were performed prior to the addition of formaldehyde, to allow inoculation of equivalent cell numbers in the stimulation assays. Three biological replicates were prepared for each isolate. RAW264.7 mouse macrophage cells stably expressing the NF-κB-dependent ELAM-luciferase reporter construct were used [55]. Cells were seeded at 2 × 10^4 ^cells/well of a 96-well flat bottomed tissue culture plate in 100 μl LPS-free media (RPMI plus 0.5 mg/ml G418, supplemented with 10% FCS and 1 mM L-glutamine) and incubated overnight at 37°C, 5% CO_2_. Cells were then stimulated with VSSA or hVISA/VISA isolates in triplicate, at a range of concentrations for 6 h at 37°C, 5% CO_2_. After removing the media 50 μl of 1 × Promega passive lysis buffer was added to each well (5 min at room temperature). Twenty μl from each well was transferred to a white opaque TC_96 _plate, and 30 μl Luciferase assay reagent (Promega) added. Luciferase activity was measured with a Fluro-Optima luminometer. The statistical significance of the data was evaluated by paired *t *test using GraphPad Prism 4.0 (GraphPad Software Inc., San Diego, CA).

### TNF-α and IL-1β immunoassays

These assays were performed using the Quantikine Mouse TNF-α and IL-1β immunoassay kits (R&D Systems) according to the manufacturers' instructions. Briefly, RAW 264.7 cells were seeded at 2 × 10^4 ^cells/well of a 96-well flat bottomed tissue culture plate in 100 μl LPS-free media (RPMI supplemented with 10% FCS and 1 mM L-glutamine) and incubated overnight at 37°C, 5% CO_2_. Cells were then stimulated with VSSA and VISA in triplicate for 6 h at 37°C, 5% CO_2_. After spinning the plates supernatant from each well was stored at -20°C prior to analysis. The statistical significance of the data was evaluated by paired *t *test using GraphPad Prism 4.0 (GraphPad Software Inc., San Diego, CA)

## Authors' contributions

BH carried out all the experimental work, except for the assay to assess innate immune responses which were performed by DS. The study was conceived by JD, PJ, BH and AM. PW carried out initial characterization of the strains used in the study. PJ assisted with statistical analysis and TS assisted with assessment of sequencing results. AM, JD and TS assisted BH with drafting the manuscript. All authors read and approved the final manuscript.

## Supplementary Material

Additional file 1 **Complete figure **1. Full image of heat map analysis of selected genes which were differentially expressed in at least 2 isolate pairs. Click here for file

Additional file 2 **Data table **1. Summary of microarray results for the hVISA/VISA to VSSA comparison. Any genes which were consistently up or down regulated across at least two clinical pairs were included in the set of "genes of interest". Data are presented as the fold ratio of hVISA/VISA to VSSA. Table 1a and 1b are without vancomycin exposure. Table 1c and 1d are after vancomycin exposure (see methods section for details). Click here for file

Additional file 3 **Global heat map**. Heat map analysis for all genes on the microarray. The results are expressed as fold ratio of gene expression for hVISA/VISA compared to VSSA. Results are presented for all 5 isolate pairs without antibiotic exposure and all 5 isolate pairs after exposure to vancomycin. Click here for file
