# Supplementary figures and images for: Different bacterial gene expression patterns and attenuated host immune responses are associated with the evolution of low-level vancomycin resistance during persistent methicillin-resistant Staphylococcus aureus bacteraemia
Source: BMC Microbiol. 2008 Feb 27;8:39. doi: 10.1186/1471-2180-8-39 (PMC2289824; doi:10.1186/1471-2180-8-39)

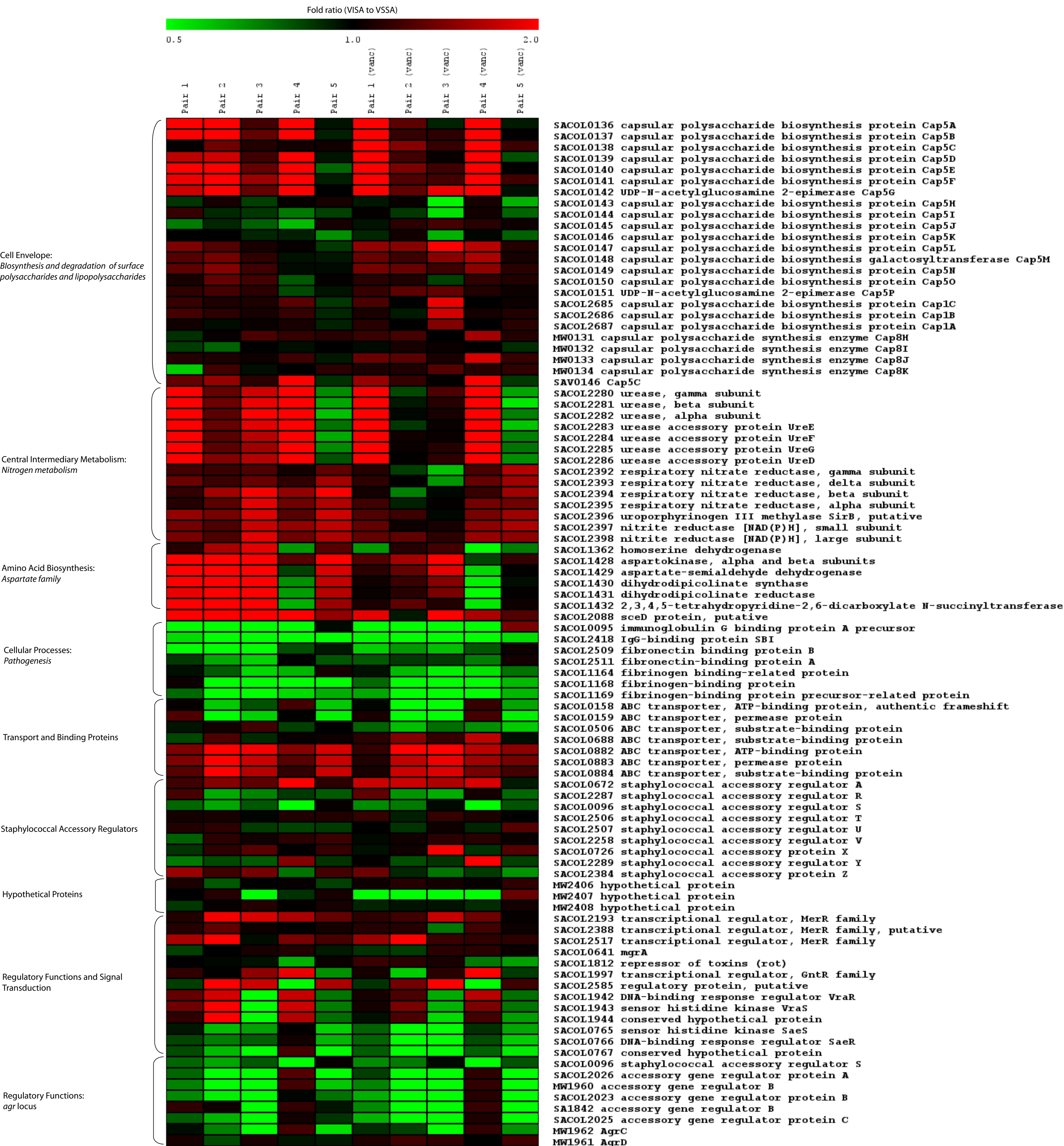

Supplement: Additional file 1 — Complete figure 1. Full image of heat map analysis of selected genes which were differentially expressed in at least 2 isolate pairs. [file 1471-2180-8-39-S1.pdf]
