# Supplementary material for: Different bacterial gene expression patterns and attenuated host immune responses are associated with the evolution of low-level vancomycin resistance during persistent methicillin-resistant Staphylococcus aureus bacteraemia
Source: BMC Microbiol. 2008 Feb 27;8:39. doi: 10.1186/1471-2180-8-39 (PMC2289824; doi:10.1186/1471-2180-8-39)
Supplement: Additional file 3 — Global heat map. Heat map analysis for all genes on the microarray. The results are expressed as fold ratio of gene expression for hVISA/VISA compared to VSSA. Results are presented for all 5 isolate pairs without antibiotic exposure and all 5 isolate pairs after exposure to vancomycin. [file 1471-2180-8-39-S3.pdf]

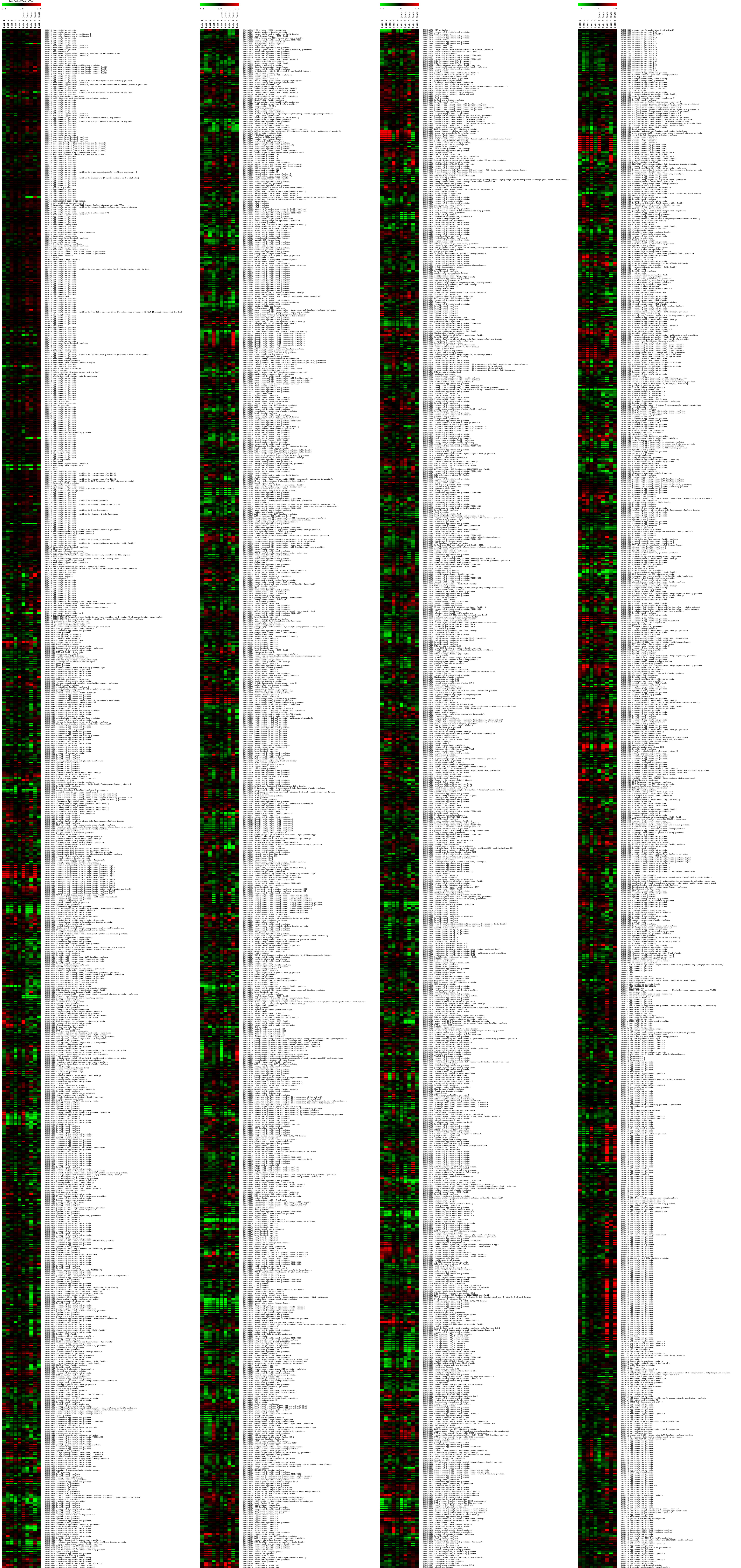
Fold Ratio (VISA to VSSA)
0.5
1.0
2.0
Pair 1
Pair 2
Pair 3
Pair 4
Pair 5
Pair 1 (vanc)
Pair 2 (vanc)
Pair 3 (vanc)
Pair 4 (vanc)
Pair 5 (vanc)
