# Supplementary material for: Different bacterial gene expression patterns and attenuated host immune responses are associated with the evolution of low-level vancomycin resistance during persistent methicillin-resistant Staphylococcus aureus bacteraemia
Source: BMC Microbiol. 2008 Feb 27;8:39. doi: 10.1186/1471-2180-8-39 (PMC2289824; doi:10.1186/1471-2180-8-39)
Supplement: Additional file 2 — Data table 1. Summary of microarray results for the hVISA/VISA to VSSA comparison. Any genes which were consistently up or down regulated across at least two clinical pairs were included in the set of "genes of interest". Data are presented as the fold ratio of hVISA/VISA to VSSA. Table 1a and 1b are without vancomycin exposure. Table 1c and 1d are after vancomycin exposure (see methods section for details). [file 1471-2180-8-39-S2.pdf]

Data table 1a. Genes differentially regulated in hVISA/VISA compared to matched VSSA, up-regulated.

			Isolate Pair				
**ORF ID** [a]	**Gene**	**TIGR description**[b]	**1**	**2**	**3**	**4**	**5**
**Amino acid biosynthesis:**							
Aspartate family							
SACOL1362	*hom*	homoserine dehydrogenase	1.15	1.66*	1.90*	0.70*	1.35
SACOL1428	*lysC*	aspartokinase, alpha and beta subunits	2.20*	2.49*	5.12*	1.34*	2.21*
SACOL1429	*asd*	aspartate-semialdehyde dehydrogenase	2.22*	2.56*	3.52*	0.87	1.86*
SACOL1430	*dapA*	dihydrodipicolinate synthase	2.25*	2.51*	3.03*	0.72*	1.77*
SACOL1431	*dapB*	dihydrodipicolinate reductase	1.92*	2.35*	3.36*	0.69*	1.72*
SACOL1432	*dapD*	2,3,4,5-tetrahydropyridine-2,6-dicarboxylate N-succinyltransferase	2.18*	2.10*	2.83*	0.65*	1.61*
Glutamate family							
SACOL0514	*gltB*	glutamate synthase, large subunit	1.23	1.79*	1.69*	1.19	1.13
Pyruvate family							
SACOL0600	*ilvE*	branched-chain amino acid aminotransferase	1.44	2.06*	2.16*	1.21*	1.24
SACOL2043	*ilvB*	acetolactate synthase, large subunit, biosynthetic type	1.57	1.95*	1.78*	1.13	1.14
SACOL2045	*ilvC*	ketol-acid reductoisomerase	1.50	1.82*	1.88*	1.09	1.30
SACOL2046	*leuA*	2-isopropylmalate synthase	1.75*	1.73	1.85*	1.16	1.19
**Biosynthesis of cofactors, prosthetic groups and carriers**:							
Glutathione and anlalogs							
SACOL2641	*gpxA2*	glutathione peroxidase	3.04*	1.97*	2.12*	1.02	1.45
Heme, porphyrin, and cobalamin							
SACOL2396		uroporphyrinogen III methylase SirB, putative	1.59	1.47	1.87*	1.34*	1.83*
**Cellular Processes:**							
Adaptions to atypical conditions							
SACOL1759		universal stress protein family	1.11	2.17*	2.54*	1.05	1.79*
SACOL2131		Dps family protein	0.93	2.04	2.76*	1.60*	1.84
Detoxification							
SACOL1368	*kataA*	catalase	1.08	1.93*	1.46	1.06	1.52*
Pathogenesis							
SACOL0270		staphyloxanthin biosynthesis protein, putative	2.14*	1.06	1.61*	0.75*	1.35
Others							
SACOL1440		xpaC protein, putative	0.99	1.53*	1.37*	1.61*	1.07
**Cell envelope:**							
Biosynthesis and degradation of murein sacculus and peptidoglycan							
SACOL0248	*lrgB*	lrgB protein	0.75	1.69*	2.08*	0.73	1.61*
Biosynthesis and degradation of surface polysaccharides and lipopolysaccharides							
SACOL0136	*cap5A*	capsular polysaccharide biosynthesis protein Cap5A	2.13*	2.04*	1.27	2.53*	0.96
SACOL0137	*cap5B*	capsular polysaccharide biosynthesis protein Cap5B	1.99	2.02*	1.39*	2.91*	0.94
SACOL0139	*cap5D*	capsular polysaccharide biosynthesis protein Cap5D	1.75	1.82*	1.25	2.05*	0.96
SACOL0140	*cap5E*	capsular polysaccharide biosynthesis protein Cap5E	2.27	2.06*	1.40*	2.64*	0.80
SACOL0141	*cap5F*	capsular polysaccharide biosynthesis protein Cap5F	1.94	2.42*	1.62*	2.29*	0.94
Other							
SACOL2539	*srtA*	sortase	1.20	1.63	1.79*	1.02	1.62*
**Central intermediary metabolism:**							
Nitrogen metabolism							
SACOL2280	*ureA*	urease, gamma subunit	3.75*	1.40	1.82*	2.69*	0.73

SACOL2281	*ureB*	urease, beta subunit	3.65	1.43	1.93*	2.78*	0.67
SACOL2282	*ureC*	urease, alpha subunit	4.96*	1.36	2.01*	3.14*	0.62*
SACOL2283	*ureE*	urease accessory protein UreE	2.95*	1.41	1.89*	3.20*	0.78
SACOL2284	*ureF*	urease accessory protein UreF	4.27*	1.36	1.73*	3.19*	0.66*
SACOL2285	*ureG*	urease accessory protein UreG	3.71*	1.36	1.63*	2.94*	0.73*
SACOL2286	*ure*D	urease accessory protein UreD	3.04*	1.51	1.72*	2.85*	0.84
SACOL2394	*narH*	respiratory nitrate reductase, beta subunit	1.20	1.64	1.97*	1.58*	1.99
SACOL2395	*narG*	respiratory nitrate reductase, alpha subunit	1.15	1.35*	1.90*	1.44*	1.70*
SACOL2397	*nirD*	nitrite reductase [NAD(P)H], small subunit	1.45*	1.30	1.79*	1.58*	1.75*
Other							
SACOL1920		D-isomer specific 2-hydroxyacid dehydrogenase family protein	1.08	1.75*	1.57*	1.01	1.30
SACOL2574		D-isomer specific 2-hydroxyacid dehydrogenase family protein	1.31	2.32*	2.23*	1.07	1.56
**DNA metabolism:**							
Replication, recombination, repair							
SACOL2562	*ogt*	methylated-DNA--protein-cysteine methyltransferase	1.14	1.58*	1.88*	1.00	1.44
Other							
SACOL0880		Toprim domain protein	1.23	1.53	1.50*	1.05	1.66*
**Energy metabolism:**							
Fermentation							
SACOL0111		acetoin reductase	1.05	1.80*	2.33*	1.34*	1.53
SACOL1984	*aldA2*	aldehyde dehydrogenase	1.04	1.77*	1.60*	1.26*	1.21
SACOL2178		alcohol dehydrogenase, zinc-containing	1.59	1.86*	2.09*	1.09	1.34
TCA cycle							
SACOL1449	*sucA*	2-oxoglutarate dehydrogenase, E1 component	1.81*	0.78	1.07	1.66*	0.89
**Fatty acid and phospholipid metabolism:**							
Biosynthesis							
SACOL1351	*cls1*	cardiolipin synthetase	1.24	1.93*	2.01*	1.24	1.65
**Protein fate:**							
Protein folding and stabilization							
SACOL2385		heat shock protein, Hsp20 family	1.53*	1.53*	1.61*	1.51*	1.32
**Protein synthesis:**							
Translation factors							
SACOL0815		ribosomal subunit interface protein	1.17	2.53*	2.10*	0.92	1.74*
Ribosomal proteins: synthesis and modificaion							
SACOL1369	*rpmG1*	ribosomal protein L33	1.10	2.24*	1.54*	0.88	1.80
tRNA aminoacylation							
SACOL0009	*serS*	seryl-tRNA synthetase	1.89*	2.42*	2.27*	1.57*	1.34
**Purines, pyrimidines, nucleosides and nucleotides:**							
2'-Deoxyribonucleotide metabolism							
SACOL0790.1		ribonucleoside-diphosphate reductase 2, NrdH-redoxin, putative	0.55*	1.59*	1.08	1.18*	1.52*
SACOL0792	*nrdE*	ribonucleoside-diphosphate reductase 2, alpha subunit	0.84	1.58*	1.54*	1.29	1.23
SACOL1622	*glyS*	glycyl-tRNA synthetase	1.15	1.64*	1.48*	1.72*	1.49*
**Regulatory Functions:**							
DNA interactions							
SACOL1997		transcriptional regulator, GntR family	1.19	1.02	1.56*	1.91*	0.71
SACOL2193		transcriptional regulator, MerR family	1.13	1.97*	1.86*	1.68*	1.40*
SACOL2517		transcriptional regulator, MerR family	1.67*	2.67*	0.97	1.47*	1.28

Other							
SACOL2585		regulatory protein, putative	0.88	2.60*	1.81*	0.51*	1.67
**Signal transduction:**							
2-component system							
SACOL1942	*vraR*	DNA-binding response regulator VraR	1.40	1.83*	0.45*	1.78*	0.77
SACOL1943	*vraS*	sensor histidine kinase VraS	1.45	2.34*	0.48*	1.82*	0.75*
**Transport and binding proteins:**							
Anions							
SACOL2386		nitrite extrusion protein	1.32	1.39	1.77*	2.08*	1.59*
Cations and iron carrying compounds							
SACOL0686		Na+/H+ antiporter, MnhG component, putative	0.85	1.62*	0.99	1.68*	0.93
SACOL2166		iron compound ABC transporter, permease protein	1.05	0.99	1.54*	1.64*	1.03
Unknown substrate							
SACOL0882		ABC transporter, ATP-binding protein	1.49	2.30*	1.97*	1.53*	1.85*
SACOL0883		ABC transporter, permease protein	1.50*	2.24*	1.80*	1.35	1.79*
SACOL0884		ABC transporter, substrate-binding protein	1.35	2.47*	1.72*	1.20	1.81*
SACOL2279		transporter, putative	2.35*	1.74	2.05*	1.65*	0.81
Other							
SACOL2348		drug transporter, putative	0.84	1.46	1.56*	1.04	1.69*
SACOL2619		amino acid permease	1.26	2.82*	1.80*	0.54*	1.58*
**Unknown Functions:**							
Enzymes of unknown specificity							
SACOL0399		oxidoreductase, putative	1.29	1.61*	1.78*	1.21*	1.32
SACOL0655		oxidoreductase, aldo/keto reductase family	1.22	1.41*	1.66*	0.65*	1.16
SACOL1543		oxidoreductase, aldo/keto reductase family	1.67*	1.70*	1.86*	1.03	1.30
SACOL1772		aminotransferase, class V	1.00	2.58*	1.56*	1.00	1.37
SACOL1774		hydrolase, haloacid dehalogenase-like family	1.25	1.60*	1.56*	1.21	1.25
SACOL2192		oxidoreductase, aldo/keto reductase family	1.53	1.98	1.86*	1.59*	1.38
SACOL2575		aminotransferase, class I	1.45	2.28*	2.08*	1.11	1.58
SACOL2620		aminotransferase, class III	1.08	3.44*	2.63*	0.45*	1.72*
General							
SACOL0089		antigen, 67 kDa	1.42	1.63*	1.54*	1.12	1.11
SACOL0220		flavohemoprotein, putative	1.25	1.91*	1.97*	0.82*	1.42*
SACOL1553		glyoxalase family protein	0.89	1.64	1.68*	1.51*	1.62*
SACOL2088		sceD protein, putative	2.65*	2.01*	3.70*	2.62*	1.58
SACOL2522		DedA family protein	1.13	1.57*	1.55*	1.37*	1.23
**Hypothetical proteins:**							
SACOL2344		hypothetical protein	1.23	2.00*	2.02*	1.18*	1.49
SAV2383		assimilatory nitrite reductase [NAD(P)H] small subunit	1.16	1.23	1.64*	1.41*	1.58*
Conserved							
SACOL0467		conserved hypothetical protein	1.42	2.54*	3.65*	1.23*	1.79*
SACOL0597		conserved hypothetical protein	1.08	1.77*	1.47*	0.89*	1.58*
SACOL0669		conserved hypothetical protein	1.05	1.51*	1.12	0.95	1.77*
SACOL1944		conserved hypothetical protein (match to 1942/43)	1.19	2.50*	0.48*	1.70*	0.79
SACOL1956		conserved hypothetical protein	1.17	1.69*	0.42*	1.76*	0.77
SACOL2550		conserved hypothetical protein	1.37	2.36*	2.12*	1.50*	1.36
SACOL2551		conserved hypothetical protein TIGR00051	1.23	2.33*	2.02*	1.86*	1.49
SACOL2710		conserved hypothetical protein	1.42	2.31*	1.66*	0.67*	1.31

Data table 1b. Genes differentially regulated in hVISA/VISA compared to matched VSSA, down-regulated.

			Isolate pair				
**ORF ID[a]**	**Gene**	**TIGR description[b]**	**1**	**2**	**3**	**4**	**5**
**Biosynthesis of cofactors, prosthetic groups and carriers:**							
Pyridoxine							
SACOL0564		pyridoxine biosynthesis protein	0.97	0.50*	0.72*	0.85	0.59*
**Cell envelope:**							
Biosynthesis and degradation of surface polysaccharides and lipopolysaccharides							
SACOL1043		glycosyl transferase, group 1 family protein	0.40*	0.36	0.70*	0.60*	0.96
Other							
SACOL2354		membrane protein, putative	0.69	0.86	0.65*	0.52*	0.95
SACOL2520		membrane protein, putative	0.76	0.68*	0.57	1.12	0.54*
**Cellular Processes:**							
Pathogenesis							
SACOL0095	*spa*	immunoglobulin G binding protein A precursor	0.12*	0.08*	0.19*	0.11*	0.98
SACOL2291		staphyloxanthin biosynthesis protein	1.24	0.37*	0.62*	1.40*	0.65
SACOL2418		IgG-binding protein SBI	0.57*	0.31*	0.21*	0.31*	0.41
SACOL2509	*fnbB*	fibronectin binding protein B	0.47*	0.37*	0.37*	0.84	0.96
SACOL2511	*fnbA*	fibronectin-binding protein A	0.89	0.66*	0.59*	1.00	0.83
Toxin production and resistance							
SACOL0478		exotoxin 3, putative	0.79	0.83	0.60*	0.66*	0.99
**DNA metabolism:**							
Replication, recombination, repair							
SACOL0678		integrase/recombinase, phage integrase family	0.69	0.60*	0.48*	1.28*	0.63*
**Energy metabolism:**							
Amino acids and amines							
SACOL2327	*hutG*	formiminoglutamase	0.54*	0.85	0.80	0.62*	0.93
ATP-proton motive force interconversion							
SACOL2101	*atpB*	ATP synthase F0, A subunit	1.27	0.66	0.66*	0.64*	0.75
**Protein fate:**							
Protein and peptide secretion and trafficking							
SACOL1251		cell division protein FtsY, putative	1.02	0.61*	0.66*	1.69*	0.81
**Protein synthesis:**							
Ribosomal proteins: synthesis and modification							
SACOL1702	*rplU*	ribosomal protein L21	0.82	0.59*	0.56*	1.02	0.79
Translation factors							
SACOL1278	*frr*	ribosome recycling factor	0.75	0.53*	0.62*	0.97	0.66*
tRNA aminoacylation							
SACOL1962	*gatC*	glutamyl-tRNA(Gln) amidotransferase, C subunit	0.83	0.70	0.39*	0.47*	0.76
tRNA and rRNA base modification							
SACOL1695	*queA*	S-adenosylmethionine: tRNA ribosyltransferase-isomerase	0.93	0.57*	0.61*	1.48*	0.75

**Purines, pyrimidines, nucleosides and nucleotides:**							
Nucleotide and nucleoside interconversions							
SACOL1277	*pyrH*	uridylate kinase	0.68	0.53*	0.46*	0.79*	0.65*
SACOL1371	*guaC*	GMP reductase	0.41*	0.45*	0.53*	1.16*	0.65
SACOL2111	*tdk*	thymidine kinase	0.93	0.47*	0.60*	1.17*	0.71
Pyrimidine ribonucleotide biosynthesis							
SACOL1214	*carA*	carbamoyl-phosphate synthase, small subunit	1.35	0.38*	0.62*	0.75	0.55
SACOL1216	*pyrF*	orotidine 5-phosphate decarboxylase	1.63	0.37*	0.63*	0.73	0.55*
Salvage of nucleosides and nucleotides							
SACOL2128	*pdp*	pyrimidine-nucleoside phosphorylase	0.78	0.59*	0.65*	0.94	0.85
**Regulatory Functions:**							
DNA interactions							
SACOL2308		phosphosugar-binding transcriptional regulator, RpiR family	0.82	0.60	0.47*	0.53*	0.83
RNA interactions							
SACOL1210	*pyrR*	pyrimidine operon regulatory protein	0.74	0.46	0.41*	0.43*	0.59
Other							
SACOL1904		transcriptional regulator, putative	0.78	0.82	0.62*	0.52*	0.80
MW1960	*agrB*	accessory gene regulator B	0.75	0.33*	0.28*	1.27	0.60*
SACOL2023	*agrB*	accessory gene regulator protein B	0.73	0.53*	0.28*	0.99	0.77
SACOL2026	*agrA*	accessory gene regulator protein A	0.80	0.34*	0.20*	1.34	0.45*
**Transport and binding proteins:**							
Amino acids, peptides and amines							
SACOL1108		spermidine/putrescine ABC transporter, ATP-binding protein	1.08	0.35*	0.51*	1.12	0.76
SACOL1110		spermidine/putrescine ABC transporter, permease protein	0.86	0.42*	0.42*	0.89	0.86
SACOL2382		proton/sodium-glutamate symport protein	1.07	0.90	0.60*	0.63*	0.81
Carbohydrates, organic alcohols and acids							
SACOL0175		PTS system, IIABC components	0.83	0.61*	0.72*	0.51*	0.85
SACOL0454		sodium:dicarboxylate symporter family protein	0.72	0.56*	0.63*	0.82	0.68*
Cations and iron carrying compounds							
SACOL0679		Na+/H+ antiporter, MnhA component, putative	0.71	1.05	0.65*	1.59*	0.66*
Nucleosides, purines, pyrimidines							
SACOL1211	*uraA*	uracil permease	0.77	0.51	0.58*	0.77	0.59*
Unknown substrate							
SACOL0159		ABC transporter, permease protein	1.31	0.47*	0.59*	0.99	0.52*
**Unknown Functions:**							
General							
SACOL0507		LysM domain protein	0.81	0.26*	0.46*	0.78*	0.54*
SACOL1164		fibrinogen binding-related protein	0.97	0.82	0.57*	0.64*	1.09
SACOL1168	*efb*	fibrinogen-binding protein	1.05	0.39	0.30*	0.45*	0.45
SACOL1169		fibrinogen-binding protein precursor-related protein	0.80	0.46	0.42*	0.57*	0.66
SACOL2591		actVA 4 protein	0.88	0.91	0.64*	0.65*	0.82
**Hypothetical proteins:**							
SAV0411		exotoxin homolog	0.85	0.61*	0.69*	0.51*	0.94
Conserved							
SACOL0157		conserved hypothetical protein	1.09	0.38*	0.41*	1.07	0.52*
SACOL0160		conserved hypothetical protein	1.20	0.59*	0.74*	0.87	0.59*
SACOL0199		conserved hypothetical protein	0.73	0.57	0.43*	0.42*	0.61
SACOL0767		conserved hypothetical protein	0.85	0.63	0.39*	1.28*	0.51*
SACOL0802		conserved hypothetical protein	1.07	0.78	0.57*	0.57*	0.71
SACOL1821		conserved hypothetical protein	1.07	0.88	0.60*	0.55*	0.89
SACOL2020		conserved hypothetical protein	0.49	0.85	1.02	0.66	1.00

SACOL2373	conserved hypothetical protein	0.56*	0.66	0.59*	0.47*	0.86
SACOL2461	conserved hypothetical protein	0.83	0.76	0.53*	0.44*	1.03
Domain						
SACOL1847	conserved domain protein, putative	0.80	0.28*	0.63*	0.71*	0.69

Data table 1c. Genes differentially regulated in hVISA/VISA compared to matched VSSA after vancomycin exposure, up-regulated.

ORF ID[a]	Gene	TIGR description[b]	Isolate pair 1	2	3	4	5
**Amino acid biosynthesis:**							
Pyruvate family							
SACOL0600	*ilvE*	branched-chain amino acid aminotransferase	1.04	2.10*	1.89*	1.07	1.17
**Biosynthesis of cofactors, prosthetic groups and carriers:**							
Glutathione and analogs							
SACOL2641	*gpxA2*	glutathione peroxidase	3.19*	1.27	2.17*	1.04	1.06
Other							
SACOL0538	*ispE*	4-diphosphocytidyl-2C-methyl-D-erythritol kinase	2.75*	0.78	0.76	1.52*	0.87
**Cell envelope:**							
Biosynthesis and degradation of surface polysaccharides and lipopolysaccharides							
SACOL0136	*cap5A*	capsular polysaccharide biosynthesis protein Cap5A	3.23*	1.37	0.93	3.80*	0.95
SACOL0137	*cap5B*	capsular polysaccharide biosynthesis protein Cap5B	2.81*	1.21	1.18	3.62*	1.01
SACOL0138	*cap5C*	capsular polysaccharide biosynthesis protein Cap5C	3.58*	1.54	1.21	4.46*	1.24
SACOL0139	*cap5D*	capsular polysaccharide biosynthesis protein Cap5D	3.52*	1.26	1.04	4.39*	0.84
SACOL0140	*cap5E*	capsular polysaccharide biosynthesis protein Cap5E	3.76*	1.50	1.25	4.88*	1.02
SACOL0141	*cap5F*	capsular polysaccharide biosynthesis protein Cap5F	2.65*	1.32	1.27	3.69*	1.26
SACOL0142	*cap5G*	capsular polysaccharide biosynthesis protein Cap5G	2.32*	1.39	1.94	3.81*	0.97
SACOL0147	*cap5L*	capsular polysaccharide biosynthesis protein Cap5L	1.59*	1.54	2.06	1.73*	1.14
SACOL1062	*atl*	bifunctional autolysin	1.62*	1.49*	1.56*	1.04	1.20
SACOL1522		elastin binding protein, putative	1.54*	1.49*	1.88*	1.10	1.31
Other							
SACOL0466		membrane protein, putative	1.42	1.59*	1.61*	0.92	1.32*
SACOL2676		LPXTG cell wall surface anchor family protein	1.87*	1.17	1.64	1.70*	1.04
**Cellular Processes:**							
Detoxification							
SACOL1368	*kataA*	catalase	1.24	1.95*	2.00*	1.36	1.55*
DNA transformation							
SACOL1003		negative regulator of competence MecA, putative	1.10	1.57*	0.99	2.42*	1.21
Pathogenesis							
SACOL0856	*clfA*	clumping factor A	2.13*	1.48	1.14	1.86*	1.02
Toxin production and resistance							
SACOL0086		drug transporter, putative	1.05	1.54*	0.99	1.57*	1.87
SACOL0672	*sarA*	staphylococcal accessory regulator A	1.80*	1.81*	1.69*	1.85*	0.95
SACOL2347		drug resistance transporter, EmrB/QacA subfamily	1.17	1.69*	1.93*	0.73	1.99*
SACOL2348		drug transporter, putative	1.11	1.28	1.97*	0.69	1.56*
**Central intermediary metabolism:**							
Amino sugars							
SACOL2145	*glmS*	glucosamine--fructose-6-phosphate aminotransferase (isomerizing)	1.89*	1.65	1.33*	1.63*	1.05
Nitrogen metabolism							
SACOL2280	*ureA*	urease, gamma subunit	3.37*	0.85	1.31	2.48*	0.68
SACOL2281	*ureB*	urease, beta subunit	2.89*	0.95	1.15	2.12*	0.55
SACOL2282	*ureC*	urease, alpha subunit	5.32*	0.98	1.10	2.76*	0.75
SACOL2283	*ureE*	urease accessory protein UreE	3.49*	0.89	1.04	2.61*	0.61*
SACOL2284	*ureF*	urease accessory protein UreF	4.39*	1.07	1.05	2.85*	0.75
SACOL2285	*ureG*	urease accessory protein UreG	3.81*	1.05	1.15	2.80*	0.77

SACOL2286	*ureD*	urease accessory protein UreD	3.19*	1.02	1.18	2.52*	0.72*
SACOL2397	*nirD*	nitrite reductase [NAD(P)H], small subunit	1.39	1.06	1.10	1.55*	1.68*
**Energy metabolism:**							
Glycolysis/gluconeogenesis							
SACOL1745	*pyk*	pyruvate kinase	1.17	1.88*	1.31	1.00	1.54*
Sugars							
SACOL2149	*mtlD*	mannitol-1-phosphate 5-dehydrogenase	1.46	1.70*	1.53*	0.84	1.29*
Other							
SACOL2553		pyruvate oxidase	1.35	2.14*	1.52*	0.76	1.53*
**Protein synthesis:**							
Ribosomal proteins: synthesis and modification							
SACOL1254	*rpsP*	ribosomal protein S16	1.12	1.15	2.18*	1.60*	1.03
tRNA aminoacylation							
SACOL0009	*serS*	seryl-tRNA synthetase	2.10*	1.84*	1.62	1.76*	0.91
**Purines, pyrimidines, nucleosides and nucleotides:**							
2'-Deoxyribonucleotide metabolism							
SACOL0791	*nrdI*	nrdI protein	0.89	1.55*	1.59	1.87*	1.29
**Regulatory Functions:**							
DNA interactions							
SACOL0539	*purR*	pur operon repressor	3.68*	0.97	0.66	1.65*	0.88
SACOL1374	*lexA*	LexA repressor	1.02	1.55*	2.09*	1.51*	1.11
SACOL2517		transcriptional regulator, MerR family	1.61*	1.99*	1.23	1.19	1.17
**Transcription:**							
Transcription factors							
SACOL2113	*rho*	transcription termination factor Rho	1.96*	1.25	1.75*	1.26	1.29*
**Transport and binding proteins:**							
Amino acids, peptides and amines							
SACOL2619		amino acid permease	1.10	2.59*	2.04*	0.58*	1.37*
Carbohydrates, organic alcohols and acids							
SACOL2148		PTS system, mannitol-specific IIA component	1.74*	2.14*	1.65*	0.83	1.36*
Unknown substrate							
SACOL0688		ABC transporter, substrate-binding protein	1.18	1.52*	1.22	1.76*	0.97
SACOL0882		ABC transporter, ATP-binding protein	1.20	2.10*	2.04*	1.74*	1.55*
SACOL0883		ABC transporter, permease protein	1.13	1.76*	1.94*	1.64*	1.47*
SACOL0884		ABC transporter, substrate-binding protein	1.10	1.80*	1.87*	1.46*	1.27*
**Unknown Functions:**							
Enzymes of unknown specificity							
SACOL0399		oxidoreductase, putative	1.49*	1.67*	1.70*	1.00	1.28
SACOL1543		oxidoreductase, aldo/keto reductase family	1.77*	1.69*	1.98*	0.98	1.41*
SACOL2321		oxidoreductase, short chain dehydrogenase/ reductase family	1.35	1.97*	1.67*	1.19	1.25
SACOL2446		epimerase/dehydratase, putative	2.19*	0.83	3.04*	1.02	1.02
SACOL2575		aminotransferase, class I	1.41	1.53*	2.42*	1.37	1.26
SACOL2597		hydrolase, alpha/beta hydrolase fold family	1.25	1.68*	2.00*	0.85	1.47*
SACOL2620		aminotransferase, class III	1.14	2.41*	4.98*	0.46*	1.44*
General							
SACOL0089		antigen, 67 kDa	2.12*	1.75*	1.29	0.88	1.14
SACOL2088		sceD protein, putative	1.91*	0.95	3.66	1.68*	1.33
**Hypothetical proteins:**							
SAV0058		hypothetical protein	1.30	2.42*	1.57*	0.85	1.41*
SAV0069		hypothetical protein	1.65*	1.28	1.02	2.20*	0.97

SAV0394	hypothetical protein	1.00	1.23	5.19*	0.86	2.74*
SAV0854	hypothetical protein	1.02	1.28	3.93*	1.06	3.11*
SAV0871	hypothetical protein	0.95	1.19	2.49*	0.95	1.54*
SAV0882	hypothetical protein	1.09	1.28	2.84*	1.08	2.94*
SAV0884	hypothetical protein	0.93	1.18	3.28*	1.02	2.40*
SAV0898	hypothetical protein	1.01	1.11	3.89*	1.01	1.76*
Conserved						
SACOL0409	conserved hypothetical protein					
SACOL0467	conserved hypothetical protein	1.50*	1.75*	2.22*	0.86	1.50*
SACOL0511	conserved hypothetical protein	1.38	1.88*	1.43	0.87	1.54*
SACOL2519	conserved hypothetical protein	1.71*	1.15	1.31	1.80*	0.67
SACOL2711	conserved hypothetical protein	1.39	1.61*	1.51*	0.79	1.10
SACOL2723	conserved hypothetical protein	1.72*	1.87*	0.87	0.69*	1.22

Data table 1d. Genes differentially regulated in hVISA/VISA compared to matched VSSA after vancomycin exposure, down-regulated.

ORF ID [a]	Gene	TIGR description [b]	Isolate pair 1	2	3	4	5
**Amino acid biosynthesis:**							
Aspartate family							
SACOL0503		trans-sulfuration enzyme family protein	0.97	0.31*	0.39*	1.65*	0.51*
Glutamate family							
SACOL0963	*argH*	argininosuccinate lyase	0.88	0.31*	0.35*	0.27*	1.17
SACOL0964	*argG*	argininosuccinate synthase	1.01	0.51*	0.38	0.41*	1.20
Serine family							
SACOL0502		cysteine synthase/cystathionine beta-synthase family protein	0.91	0.29*	0.45*	1.44*	0.56*
**Biosynthesis of cofactors, prosthetic groups, and carriers:**							
Pyridoxine							
SACOL0564		pyridoxine biosynthesis protein	0.87	0.65*	0.44*	0.78	0.71*
**Cell envelope:**							
Biosynthesis and degradation of murein sacculus and peptidoglycan							
SACOL2116	*murAB*	UDP-N-acetylglucosamine 1-carboxyvinyltransferase 2	0.81	0.63*	0.57*	0.98	0.73*
Biosynthesis and degradation of surface polysaccharides and lipopolysaccharides							
SACOL1043		glycosyl transferase, group 1 family protein	0.40*	0.51*	0.81	0.49	0.83
SACOL2103		UDP-N-acetylglucosamine 2-epimerase	0.97	0.53	0.09*	1.65	0.35*
Other							
SACOL0479		surface protein, putative	0.94	0.55*	0.91	0.41*	0.80
SACOL1168	*efb*	fibrinogen-binding protein	0.79	0.44*	0.34*	0.39*	0.63
SACOL2451		amino acid ABC transporter, amino acid-binding protein	0.95	0.62*	0.54*	1.13	0.70
SACOL2520		membrane protein, putative	1.19	0.67*	0.66	1.16	0.58*
SACOL2694	*geh*	lipase	1.20	0.65*	0.59	1.11	0.44*
**Cellular Processes:**							
Pathogenesis							
SACOL0095	*spa*	immunoglobulin G binding protein A precursor	0.07*	0.13*	0.25*	0.08*	1.49
SACOL2418		IgG-binding protein SBI	0.51*	0.32*	0.39*	0.35*	0.56*
Toxin production and resistance							
SACOL0478		exotoxin 3, putative	0.70	0.36*	1.44	0.46*	0.74
SACOL1173	*hlY*	alpha-hemolysin precursor	1.04	0.40*	0.66	0.62*	0.81
SACOL1475		drug transporter, putative	0.49	0.60*	1.15	0.46*	1.38
SACOL2004		leukocidin F subunit precursor, putative	0.77	0.27*	0.38*	0.29*	0.48*
SACOL2006		Aerolysin/Leukocidin family protein	0.88	0.44*	0.76	0.39*	0.70
SACOL2523		drug transporter, putative	1.06	0.75	0.54*	0.66*	0.75*
SACOL2524		transcriptional regulator, MarR family	0.85	0.51*	0.48*	0.68	0.55*
**DNA metabolism:**							
Replication, recombination, repair							
SACOL0678		integrase/recombinase, phage integrase family	0.70	0.38*	0.54*	1.16	0.46*
**Energy metabolism:**							
Amino acids and amines							
SACOL1477	*ilvA1*	threonine dehydratase, catabolic	0.48	0.47*	0.80	0.26*	1.80
SACOL1478	*ald1*	alanine dehydrogenase	0.49	0.49*	0.71	0.25*	1.26

SACOL1593		glycine cleavage system P protein, subunit 2	0.99	0.59*	0.57*	0.82	0.82
SACOL2654	*arcC2*	carbamate kinase	1.15	0.66*	0.89	0.48*	0.97
Anaerobic							
SACOL0204	*pflB*	formate acetyltransferase	0.47	0.28*	0.21*	0.29*	0.71*
SACOL0205	*pflA*	pyruvate formate-lyase-activating enzyme	0.51	0.28*	0.22*	0.35*	0.63*
ATP-proton motive force interconversion							
SACOL2096	*atpG*	ATP synthase F1, gamma subunit	0.97	0.58	0.59*	0.58*	0.82
SACOL2097	*atpA*	ATP synthase F1, alpha subunit	1.08	0.58	0.57*	0.63*	0.91
SACOL2099	*atpF*	ATP synthase F0, B subunit	1.11	0.58	0.55*	0.64*	0.87
SACOL2100	*atpE*	ATP synthase F0, C subunit	1.06	0.46*	0.67*	0.65*	0.93
SACOL2101	*atpB*	ATP synthase F0, A subunit	1.22	0.54	0.54*	0.63*	0.93
Biosynthesis and degradation of polysaccharides							
SACOL2183	*lacD*	tagatose 1,6-diphosphate aldolase	0.25*	0.47*	0.91	0.92	0.60
Electron transport							
SACOL0494	*nuoF*	NADH dehydrogenase I, F subunit	0.88	0.50*	0.64	0.51*	0.92
Fermentation							
SACOL0660		alcohol dehydrogenase, zinc-containing	0.70	0.68	0.57*	0.50*	1.02
TCA cycle							
SACOL1159	*sdhA*	succinate dehydrogenase, flavoprotein subunit	0.90	0.61*	0.48*	0.78	0.65*
SACOL1262	*sucC*	succinyl-CoA synthase, beta subunit	1.13	0.56*	0.59*	0.85	0.69
SACOL1448	*sucB*	2-oxoglutarate dehydrogenase, E2 component, dihydroipoamide succinyltransferase	1.39	0.55	0.42*	0.89	0.54*
SACOL1449	*sucA*	2-oxoglutarate dehydrogenase, E1 component	1.55*	0.60*	0.53*	1.11	0.64
**Protein fate:**							
Protein folding and stabilization							
SACOL1638	*grpE*	heat shock protein GrpE	0.81	0.66*	0.76	0.70*	0.57*
SACOL2016	*groEL*	chaperonin, 60 kDa	0.56*	0.83	0.68*	0.64*	0.70*
SACOL2017	*groES*	chaperonin, 10 kDa	0.52*	0.61*	0.73	0.70*	0.57*
**Protein synthesis:**							
tRNA and rRNA base modification							
SACOL1962	*gatC*	glutamyl-tRNA(Gln) amidotransferase, C subunit	0.63*	0.72	0.89	0.57*	0.89
Translation factors							
SACOL1278	*frr*	ribosome recycling factor	0.64*	0.54*	0.61*	0.84	0.70*
**Purines, pyrimidines, nucleosides and nucleotides:**							
Nucleotide and nucleoside interconversions							
SACOL1277	*pyrH*	uridylate kinase	0.61*	0.61	0.59*	0.74	0.79
Pyrimidine ribonucleotide biosynthesis							
SACOL1212	*pyrB*	aspartate carbamoyltransferase	0.46*	0.33*	0.94	0.62	0.50*
SACOL1213	*pyrC*	dihydroorotase	0.46*	0.44*	0.73	0.92	0.65*
SACOL1214	*carA*	carbamoyl-phosphate synthase, small subunit	0.54*	0.61	0.74	0.84	0.51*
SACOL1215	*carB*	carbamoyl-phosphate synthase, large subunit	0.59	0.73	0.58*	0.88	0.43*
Salvage of nucleosides and nuceotides							
SACOL2128	*pdp*	pyrimidine-nucleoside phosphorylase	0.76	0.47*	0.48*	0.66*	0.70
**Regulatory Functions:**							
DNA interactions							
SACOL0518		transcriptional regulator, GntR family	0.98	1.13	0.65*	0.62*	0.99
SACOL1639	*hrcA*	heat-inducible transcription repressor HrcA	0.83	0.80	0.64*	0.78	0.58*
RNA interactions							
SACOL1210	*pyrR*	pyrimidine operon regulatory protein	0.58	0.39*	1.29	0.37*	0.64
Other							
SACOL1398		transcriptional regulator, putative	0.79	1.28	0.51*	0.48*	0.63*
MW1960	*agrB*	accessory gene regulator B	0.74	0.45*	0.15*	1.20	0.42*
SA1842	*agrB*	accessory gene regulator B	0.73	0.45*	0.15*	1.22	0.40*
SACOL2023	*agrB*	accessory gene regulator protein B	0.65*	0.31*	0.10*	1.15	0.29*
SACOL2025	*argC2*	accessory gene regulator protein C	0.64	0.35*	0.10*	1.12	0.31*

SACOL2026	*agrA*	accessory gene regulator protein A	0.88	0.49*	0.15*	1.18	0.37*
**Signal transduction:**							
2-component system							
SACOL0765	*saeS*	sensor histidine kinase	0.81	0.50*	0.44*	0.90	0.67
SACOL0766	*saeR*	DNA-binding response regulator	0.80	0.39*	0.37*	0.85	0.60*
**Transport and binding proteins:**							
Amino acids, peptides and amines							
SACOL1110		spermidine/putrescine ABC transporter, permease protein	0.70	0.46*	0.52*	0.81	1.08
SACOL1111		spermidine/putrescine ABC transporter, spermidine/putrescine-binding protein	0.82	0.57*	0.58*	0.52*	0.85
SACOL1476		amino acid permease	0.43*	0.41*	0.65	0.27*	1.32
SACOL1915		amino acid ABC transporter, ATP-binding protein	0.95	0.50*	0.46	0.37*	1.08
SACOL1916		amino acid ABC transporter, permease/substrate-binding protein	0.82	0.38*	0.38*	0.38*	0.98
SACOL2452		amino acid ABC transporter, permease protein	0.89	0.57*	0.57*	1.02	0.59*
SACOL2453		amino acid ABC transporter, ATP-binding protein	0.85	0.61*	0.60*	0.95	0.63*
Carbohydrates, organic alcohols and acids							
SACOL0175		PTS system, IIABC components	0.85	0.39*	0.43*	0.31*	0.84
SACOL0454		sodium:dicarboxylate symporter family protein	0.55*	0.58*	0.52*	0.61*	0.95
SACOL2181	*lacE*	PTS system, lactose-specific IIBC components	0.27*	0.63*	0.79	0.86	0.89
SACOL2363		L-lactate permease	0.85	0.78	0.57*	0.40*	0.81
SACOL2376		PTS system, sucrose-specific IIBC components, putative	1.17	0.64*	0.38*	0.62*	0.61*
SACOL2552		PTS system, IIABC components	0.99	0.45*	0.35*	0.41*	0.58*
Cations and iron carrying compounds							
SACOL0946		Na+/H+ antiporter family protein	0.70	0.48*	0.57	0.45*	0.72
Nucleosides, purines, pyrimidines							
SACOL1211	*uraA*	uracil permease	0.55*	0.36*	0.73	0.79	0.77
Other							
SACOL0303		acid phosphatase5-nucleotidase, lipoprotein e(P4) family	0.80	0.52*	0.62*	0.39*	0.82
Unknown substrate							
SACOL0158		ABC transporter, ATP-binding protein, authentic frameshift	0.99	0.41*	0.46*	1.21	0.67
SACOL0159		ABC transporter, permease protein	1.10	0.41*	0.26*	1.27	0.53*
SACOL0506		ABC transporter, substrate-binding protein	0.81	0.62*	0.78	0.72*	0.60*
**Unknown Functions:**							
Enzymes of unknown specificity							
SACOL0569		ATP:guanido phosphotransferase family protein	0.99	0.72	0.81	0.63*	0.61*
General							
SACOL0276	*yukA*	diarrheal toxin	1.77*	0.58*	0.68	0.56*	0.98
SACOL0507		LysM domain protein	0.72	0.57*	0.51*	0.72	0.87
SACOL0772		exsB protein	0.89	0.63*	1.07	0.54*	1.35
SACOL1164		fibrinogen binding-related protein	0.97	0.63	0.52*	0.55*	0.77
SACOL1169		fibrinogen-binding protein precursor-related protein	0.67	0.43*	0.40*	0.43*	0.64
SACOL2203		ClpA-related protein	0.89	0.64	0.12*	1.56	0.33*
SACOL2484		alkylhydroperoxidase, AhpD family	1.26	0.49*	0.75	0.83	0.52*
SACOL2584	*isaA*	immunodominant antigen A	0.84	0.36*	0.89	0.56*	0.76
**Hypothetical proteins:**							
MW1884		hypothetical protein	0.58*	0.63*	0.78	1.25	0.93
MW2407		hypothetical protein	0.05*	0.12*	0.21*	0.05*	1.41
SACOL0272		hypothetical protein	1.43	0.40*	0.64	0.47*	0.82
SACOL1846		hypothetical protein	0.99	0.17*	0.45*	0.40*	0.48*
SAV0411		exotoxin homolog	0.82	0.44*	0.95	0.60*	0.68*

SAV1425	amino acid permease homolog	0.48	0.52*	0.69	0.43*	1.08
Conserved						
SACOL0157	conserved hypothetical protein	1.15	0.24*	0.21*	1.07	0.30*
SACOL0160	conserved hypothetical protein	1.04	0.42*	0.34*	1.12	0.56*
SACOL0271	conserved hypothetical protein	3.37*	0.56	0.65*	0.29*	0.61*
SACOL0495	conserved hypothetical protein	0.74	0.37*	0.47*	0.45*	0.83
SACOL0565	conserved hypothetical protein	0.86	0.68*	0.47*	0.76	0.65*
SACOL0625	conserved hypothetical protein	0.62	1.12	0.52*	0.96	0.56*
SACOL0710	conserved hypothetical protein	0.73	0.75	0.37*	1.42*	0.64*
SACOL0767	conserved hypothetical protein	0.81	0.26*	0.27*	0.81	0.54*
SACOL0768	conserved hypothetical protein	0.76	0.33*	0.34*	0.73	0.38*
SACOL1481	conserved hypothetical protein	0.89	0.62*	1.21	0.45*	1.15
SACOL1847	conserved hypothetical protein, putative	0.91	0.26*	0.54	0.36*	0.51*
SACOL2034	conserved hypothetical protein	0.83	0.49*	0.58*	1.06	0.78
SACOL2464	conserved hypothetical protein	0.61*	0.71	0.87	1.29	0.65*
SAV0040	conserved hypothetical protein	0.77	0.76	0.61*	0.81	0.67*
SAV2510	conserved hypothetical protein	0.49*	0.82	0.93	0.41*	1.19

[a]Prefix SACOL, MRSACOL gene ID; SA, N315 gene ID; MW, MW2 gene ID; SAV, Mu50 gene ID.
[b]The Institute for Genomic Research gene function category. Note. *denotes P-value for fold change <0.05
